# Host miRNAs regulate *Escherichia coli* O157 mucosal colonization through host-mucosa-attached microbiota interactions in calves

**DOI:** 10.1186/s40168-025-02184-w

**Published:** 2025-10-23

**Authors:** Zhe Pan, Yanhong Chen, Mi Zhou, Tim A. McAllister, Tom N. McNeilly, Le Luo Guan

**Affiliations:** 1 Department of Agricultural, Food and Nutritional Science, University of Alberta, Edmonton, AB Canada; 2 APC Microbiome Ireland, University College Cork, Cork, Ireland; 3 Faculty of Land and Food Systems, The University of British Columbia, Vancouver, BC Canada; 4 Agriculture and Agri-Food Canada, Lethbridge Research Centre, Lethbridge, AB Canada; 5 Moredun Research Institute, Penicuik, UK

**Keywords:** Multi-omics, Transcriptome, MiRNAome, MiRNA profiling, Microbiota, Mucosa-attached microbiota, *Escherichia coli* O157, Stx2a, Niche breadth, Toll-like receptor

## Abstract

**Introduction:**

Host responses to pathogen colonization are central to understanding host homeostasis dynamics. Here, we used Shiga toxin (Stx)-producing *Escherichia coli* (STEC) O157 as an example to illustrate how pathogen colonization alters host-microbiome interactions and stimulates host responses. The STEC O157 is a critical foodborne pathogen, and cattle are the major asymptotic carrier with rectal anal junction (RAJ) being the major colonization site, leading to the transmission of this organism through the production chain. Therefore, this study leverages the multi-omics to evaluate host mechanisms to STEC O157 and to illustrate how mucosa-attached microbiome together with host miRNAs respond to the colonization of STEC O157.

**Results:**

The calf model was orally challenged with *E*. *coli* O157 with and without Stx2a during the 30-day trial. Mucosa-attached microbiome analysis revealed that mucosal *E*.* coli* O157 colonization limited niche occupancy of mucosa-attached microbiota regardless of the presence or absence of Stx2a. The production of Stx2a did not induce proper local host mRNA responses but miRNA profiles were more responsive to this virulent factor during high fecal shedding. The shift of toll-like receptor (TLR) expressions together with Stx2a production possibly underlined varied miRNAome-mucosa-attached microbiota interactions. For instance, during the high fecal shedding, the increased expression of *TLR2* promoted bta-miR-181b mediated host functionality, a response that was possibly blocked by Stx2a. Decreased fecal O157 shedding promoted activation of *TLR4*-stimulated host responses, which were coregulated by multiple miRNAs (i.e. bta-miR-146a and-184) and mucosa-attached microbes.

**Conclusion:**

Host mechanisms regulating STEC O157 colonization are complex interplay among mucosa-attached microbiota and host miRNAs where virulence factors could modulate such crosstalk and cause differential host responses, highlighting the importance of host-microbiome-pathogen virulence factor interactions for pathogen colonization process.

Video Abstract

**Supplementary Information:**

The online version contains supplementary material available at 10.1186/s40168-025-02184-w.

## Background

Bacterial pathogens adopt multiple strategies to colonize the gut epithelium, through complex interactions among the pathogen, gut microbiota, and host [1, 2]. The colonization of bacterial pathogens typically occurs at the intestinal mucus layer composed of a gel layer consisting of water, electrolytes, lipids, and proteins [1, 3, 4], and the bacterial invasions can be modulated by functional molecules produced by host-specific cells [1, 5]. For instance, B cells secrete immunoglobin A (IgA) into mucus that can protect the intestinal epithelium from colonization by enteric pathogens such as *Salmonella* through immune exclusion [6]. Nonetheless, some bacteria can bypass such defensive systems and colonize the epithelium in some animal systems. For example, Shiga toxin-producing *Escherichia coli* (STEC) O157 primarily colonizes at the rectal-anal junction (RAJ) of cattle without exhibiting symptoms (asymptomatic reservoir) and shed into the ambient environment and food chain to cause human illness [3, 7–9].

Previous examination of fecal microbiota showed variations in richness and diversity between super-shedders (cattle shed more than 10^4^*E*. *coli* O157 CFU/g, SS) and non-shedders [10]. However, those studies have neglected the *E*. *coli* O157 interact with both the host and the commensal microbiota directly [11] during its colonization at the epithelium. Also, gut mucosal attached commensals (differing from the lumen-associated organisms [12]) form a stable community that resists the invasion of non-native bacteria and colonization of pathogens through the direct production of inhibitors and indirect mechanisms (i.e., production of glycosylation) [13]. Without successful mucosa colonization, *E*.* coli* O157 could pass through the gut with limited host responses to its colonization. Recent studies observed that mucosa-attached microbial compositions and functions at RAJ shifted after *E*. *coli* O157 colonization [1, 3, 14, 15]. The interactions between *E*. *coli* O157 and mucosa-attached microbiota at RAJ were subjected to the production of the virulence factor Shiga toxin 2 subtype a (Stx2a) in an experimentally challenged calf model [3]. Yet, mechanisms of mucosa-attached microbiota confer *E*. *coli* O157 colonization at the RAJ are missing.

Recent studies have revealed that host immune responses are triggered during mucosal *E*. *coli* O157 colonization and a close interplay between mucosa-attached microbiota and host gene expressions [3]. Host gene expressions can be regulated by non-coding RNAs, especially microRNAs (miRNAs) [16, 17]. The identified differential expressed miRNAs along the bovine gastrointestinal tract (primarily at distal jejunum and rectum mucosa) between SS and non-shedders suggested that they may play a regulatory role in both immune (i.e., immune cell trafficking) and non-immune functions (i.e. tissue development) that could influence *E*. *coli* O157 supper-shedding [16]. Recent studies revealed that fecal miRNAs (miR-515, miR-1226) produced by gut epithelial cells can enter bacteria such as *E*. *coli* to regulate bacterial gene expressions and affect growth in mice [18], highlighting their regulatory role in host-microbiome interactions. Nonetheless, if and how host miRNAomes regulate mucosa-attached microbiota during pathogen colonization is unclear because previous studies only focused on fecal microbiota. We hypothesized that host microRNAs could mediate the interplay among mucosal-attached microbiota and host transcriptome during the *E*. *coli* O157 colonization in cattle and such a regulatory role is driven by Shiga toxin 2a (the major virulence factor in *E*. *coli* O157 [19]). Stx2a is the most potent Shiga toxin subtype and is more strongly associated with severe human disease than other Stx variants such as Stx2c [19]. In this study, we used a previously reconstructed isogenic RE strain expressing Stx2a to assess the functional impact of this virulence factor on host–microbiota interactions. Revealing host transcriptomic and miRNAomic responses are central to mechanisms of mucosa-attached microbiota to prevent *E*. *coli* O157 colonization. Therefore, this study aimed to investigate mucosal *E*. *coli* O157 colonization mechanisms as influenced by Stx2a, by deciphering host transcriptomic and miRNAomic responses in a calf challenge model.

## Results

### Study design

**Fig. 1:**
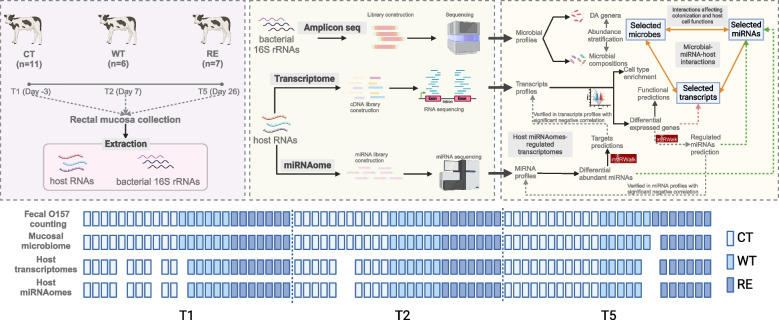
Study design and analysis flow chart. Two strains were used in this calf trial. The wild-type O157 strain contained both stx2a and stx2c prophages but only expressed stx2c (PT21/28^stx2a-stx2c+^, WT) and lab-reconstructed O157 strain contained and expressed both stx2a and stx2c prophages (RE21/28^stx2a+stx2c+^, RE). A total of 24 Holstein-Friesian veal calves were adopted at three weeks of age and randomly assigned into three groups: WT (n=6), RE (n=7), control (CT, n=11). They were orally challenged by orogastric intubation with ~10^9^CFU of each strain in 10 mL of lysogeny broth (for CT, an equal amount of broth with saline was used). The STEC O157 fecal counting was performed from pre- (To ensure calves were not colonized by STEC O157 before the challenge) to post-challenge. RAJ mucosa samples were collected from CT, WT, and RE groups 3 days before challenge (T1), 7 days (T2), and 26 days post-challenge (T5) and were subjected to host transcriptome miRNAomes, and active mucosa-attached microbial profiling. The missing rectangles in the bottom panel represent samples were missed/omitted due to limited amount/ low quality of samples for processing

To achieve this goal, two STEC O157 strains were used in this calf challenge trial: the wild-type O157 (PT21/28^stx2a−stx2c+^, WT) incapable of producing Stx2a and an isogenic lab-reconstructed Stx2a-producing O157 (RE21/28^stx2a+stx2c+^, RE) strain. The 24 Holstein–Friesian veal calves at 3 weeks of age were randomly assigned into three groups: WT (*n* = 6), RE (*n* = 7), and control (CT, *n* = 11). RAJ mucosa samples were collected from CT, WT, and RE groups at three key timepoints corresponding to critical stages of colonization: 3 days before challenge (Day − 3, T1), 7 days post-challenge (Day 7, higher fecal shedding, T2), and 26 days post-challenge (Day 26, decreased shedding, T5). Samples collected from T3 and T4 were not included in this study. A total of 71, 66, and 66 samples were used for the assessment of active mucosa-attached microbiome, host transcriptome, and host miRNAomes, respectively (Fig. 1).

### Niche occupation and functional variations as adaption traits for active mucosa-attached microbiota conferring E. coli O157 colonization independent of Stx2a production

**Fig. 2:**
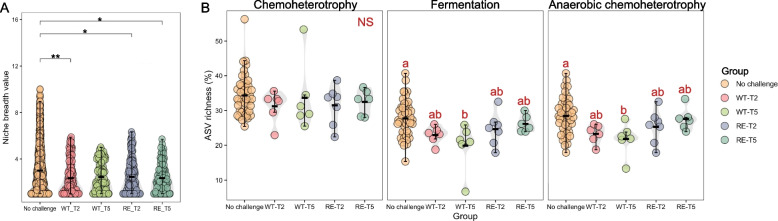
Evaluation of mucosa-attached microbiota responses to *E*. *coli* O157 colonization in calves without or post-challenge. Data from all calves across the CT, WT, and RE groups at the pre-challenge timepoint (T1) were pooled together, as they had not yet been inoculated and were microbiologically indistinguishable **A.** The Beeswarm plot showing absolute niche breadth for mucosa-attached microbes from calves without challenge or post challenge at T2 or T5 for both WT and RE (0.01 ≤ *P.*< 0.05**, 0.05 ≤ *P*< 0.1*). **B**. The Beeswarm plot showing the percentage of ASVs involved in the most enriched bacterial functions for microbial communities from calves without challenge or post challenge at T2 or T5 for both WT and RE

The niche occupancy was defined based on the niche breadth index, which indicates the diversity of recourses occupied by an individual (or species) within a certain environment [20]. A significantly higher absolute niche occupancy was identified for active mucosa-attached microbial communities in calves pre-challenge at T1 for WT and RE as compared to post-challenge calves at T2 and T5 for WT and RE (Fig. 2A). What is more, a total of three predicted bacterial functions including chemoterotrophy, fermentation, and anaerobic chemoterotrophy were highly enriched in mucosa-attached microbiota in calves from T1 to T5 among CT, WT, and RE (FAPROTAX package in R, Fig. S1). The number of bacteria possessing fermentation and anaerobic chemoterotrophy functions was decreased post-challenge for WT and RE (*P* < 0.05, Fig. 2B).

The microbial genera were stratified into abundant (≥ 1%), intermediate (≥ 0.01% and < 1%), and rare (< 0.01%) taxa based on their relative abundance [21–23]. We identified that 78–90% of mucosa-attached bacteria were designated as intermediate (Table S1), and the proportion of bacteria in abundant, intermediate, and rare categories was not affected by challenge or time (*P* > 0.1, Table S1). However, the absolute niche occupancy of both abundant and intermediate bacteria post-challenge (T2 and T5) for WT and RE was decreased in comparison with calves without challenge (Fig. S2A). The relative niche occupancy (defined as the sum of absolute niche breadth value for abundant-specific bacteria/the sum of niche breadth value for all bacteria for each group) of intermediate bacteria contributed to enriched functions for both WT and RE changed from 28% (WT-T2) to 44% (WT-T5) and 29% (RE-T2) to 38% (RE-T5), respectively (Fig. S2B). Furthermore, 82% (RE-T5) to 98% (WT-T5) bacteria belonged to intermediate taxa were involved in the most enriched bacterial functions post-challenge for WT and RE (chemoterotrophy, fermentation, and anaerobic chemoterotrophy, Fig. S2C). And 42.5% and 42.9% of intermediate bacteria were shared between WT-T2 vs. WT-T5 and RE-T2 vs. RE-T5, respectively (Fig. S2D, E). Yet only a range of 0 to 18% of bacteria belonging to either abundant or rare bacteria constituted enriched bacterial functions post-challenge for WT and RE (Fig. S2C).

What is more, out of a total of 14 differential abundant (DA) bacteria in WT compared to CT at T2, eight of them belonged to intermediate bacteria (Fig. S3A). Besides, five out of eight DA genera belonged to intermediate bacteria at WT-T5 compared to that at CT-T5 with the remaining three belonging to abundant bacteria (Fig. S3B). For RE, all seven DA microbes at T2 and *Turicibacter* at T5 were intermediate bacteria (Fig. S3C, D).

### Host transcriptomes responded to the STEC O157 colonization in a stx2a-dependent manner

An average of 33.7 ± 0.88 M high-quality mapped reads were generated from 49 ± 12 M pair-ended reads with 68.6 ± 0.06% mapping rates. A range of 18,574 to 20,445 transcripts was identified with no remarkable differences in the quantity of identified transcripts from pre- to post-challenge in CT, WT, and RE (Fig. S4).

**Fig. 3:**
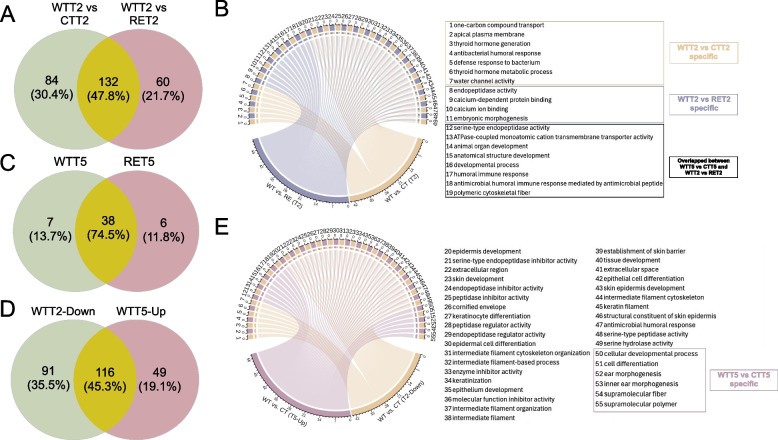
Host transcriptomic responses to the strain-specific STEC O157 colonization **A**. The Venn plot showing shared and specific differential expressed genes (DEGs) in comparison of WT-T2 vs. CT-T2 and WT-T2 vs. RE-T2. **B**. The Circos plot showing shared and specific gene ontology (GO) functions by DEGs in comparison of WT-T2 vs. CT-T2 and WT-T2 vs. RE-T2. The squares differed in color refer to specific functions for WT-T2 vs. CT-T2 (from 1 to 7) and WT-T2 vs. RE-T2 (from 8 to 11), respectively. The GO terms from 12 to 19 were specific to both WTT5 vs. CTT5 and WTT2 vs. RET2. **C**. The Venn plot showing shared and specific GO functions in comparison between WT-T5 vs. CT-T5 (WTT5) and RE-T5 vs. CT-T5 (RET5). **D**. The Venn plot showing shared and specific DEGs in comparison between WT-T2 downregulated and WT-T5 upregulated DEGs. **E**. The Circos plot showing shared and specific GO functions enriched by WT-T2 downregulated and WT-T5 upregulated DEGs. The GO terms from 50 to 55 were specific to WTT5 vs. RET5

Differentially expressed genes (DEGs, log_2_ fold change > 1.5 and Benjamini–Hochberg adjusted *P* value < 0.05) and gene ontology (GO) enrichments were identified in the comparison of WT vs. CT, RE vs. CT, and WT vs. RE post-challenge at T2 and T5. Compared to CT-T2, a total of 216 DEGs (207 downregulated DEGs enriched 37 GO terms and 9 upregulated DEGs without enriched GO terms) were identified at WT-T2, while no DEGs were identified at RE-T2. A total of 132 DEGs were shared between the comparison of WT vs. CT and WT vs. RE at T2 with 84 DEGs specific to WT vs. CT and 60 DEGs specific to WT vs. RE (Fig. 3A). Seven and 12 GO terms were specific to WT vs. CT and WT vs. RE at T2, respectively (Fig. 3B). At T5, 170 DEGs (165 upregulated DEGs enriched 42 terms and 5 downregulated DEGs enriched three GO terms related to long chain fatty acid activity) for WT vs. CT and 182 DEGs (177 upregulated DEGs enriched 44 terms and 5 downregulated DEGs without enriched terms) for RE vs. CT were identified. A total of 38 GO terms were shared between WT-T5 and RE-T5 with seven and six GO terms specific to WT-T5 and RE-T5, respectively (Fig. 3C). A sum of 116 DEGs and 30 GO terms were shared between downregulated DEGs at WT-T2 (T2 Down) and upregulated DEGs at WT-T5 (T5 Up) with seven and 14 GO terms specific T2 Down and T5 Up, respectively (Fig. 3D, 3E). Among identified GO terms, defense response to bacterium, humoral immune response, antimicrobial and antibacterial humoral response, and antimicrobial humoral immune response mediated by antimicrobial peptide were directly related to host immunity with 16 DEGs involved (Table S2). Furthermore, networks showed that DEGs enriched GO terms were clustered into two modules with functions related to extracellular region and barrier integrity in WT at T2 (Fig. S5A) and T5 (Fig. S5B).

### Host miRNA profiles were more sensitive to stx2a production than STEC O157 colonization

For host miRNAomes, an average of 6.6 ± 3.0 M mapped reads were generated from 8.2 ± 4.0 M reads with an average mapping rate of 79.3%. A range of 406 ± 60 (CT-T1) to 436 ± 19 (CT-T5) expressed host miRNAs were identified with no significant differences in terms of CT, WT, and RE from T1 to T5 (Table S3).

No differential expressed (DE, absolute log_2_ fold change > 1.5 and false discovery rate adjusted *P* < 0.05) host miRNAs were identified at WT-T2 compared to CT-T2. However, two upregulated miRNAs (bta-miR-219 and −1224) were identified at RE-T2 compared to CT-T2 (Table S4, S5). Compared to miRNAs detected at CT-T5, a total of five (four upregulated bta-miR-184, −2311, −2887, and −2440; one downregulated bta-miR-211) miRNAs were identified for WT-T5 and no DE miRNAs for RE-T5 (Table S4, S5). Compared to miRNAs detected in RE, a total of three (two upregulated bta-miR-101 and −142-3p; one downregulated bta-miR-760-3p), fourteen (one upregulated bta-miR-2285bh; 13 downregulated bta-miR-744, −874, −6517, −10,179-5p, −11,976, −3957, −10,167-3p, −485, −10,182-5p, −296-3p, −502b, −423-5p, −2474, −760-3p, −3533, −504, −1343-3p, −11,982, −1307, −1291, −365-5p, −2440, −10,185-5p, −1224, and −11,980), and six (one upregulated bta-miR-2311; five downregulated bta-miR-181c, −362-3p, −2285f, −2285ce, and −2285bf) DE miRNAs were identified at T1, T2, and T5 in WT, respectively (Tables S4, S5).

### Patterns of miRNAome-regulated host functionsrelated to strain-specific STEC O157 colonization

The enriched functions of DEGs which were negatively correlated with miRNAome (Pearson *R* < − 0.99 and *P*_adj_ < 0.01) were identified for both WT and RE post-challenge (T2 and T5, Fig. S6). The patterns of miRNAome-regulated host functions were defined based on the quantity of miRNAs associated with each function. Multiple miRNAs regulating the same functions were defined as functional redundancy [24], and the ability of one miRNA that regulates multiple functions was defined as functional compensation [25] in the current study.

**Fig. 4:**
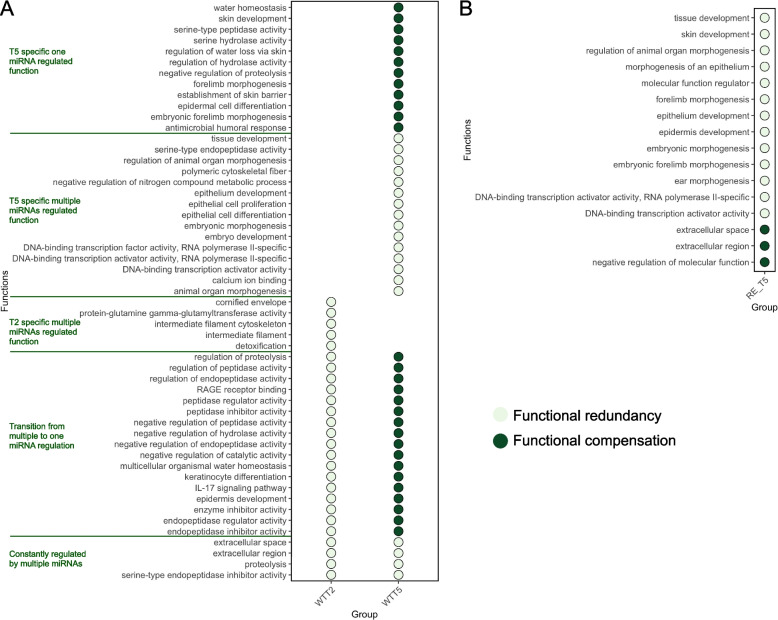
Patterns of miRNAome regulated host functions for WT (**A**) and RE (**B**). The color of each dot refers to the patterns of miRNA regulating host functions (redundancy or compensation). The functional redundancy refers to multiple miRNAs regulating the same functions and the ability of one miRNA that regulate multiple functions is defined as functional compensation

A total of 26, 48, and 16 host functions were identified to be regulated by miRNAomes at WT-T2, WT-T5, and RE-T5, respectively (Fig. S6). For WT, all 26 functions at T2 and 19 functions at T5 were regulated by multiple miRNAs (functional redundancy); however, only four functions (extracellular space, extracellular region, proteolysis, and serine-type endopeptidase inhibitor activity) were consistently regulated by multiple miRNAs at both T2 and T5 (Fig. 4A). Particularly, bta-let-7i, bta-miR-181b, and bta-miR-2431.5p were mostly coordinatively regulated host functions at WT-T2 (Fig. S7A). Nonetheless, a total of 17 functions shifted the regulatory pattern from redundancy to compensation that was initially regulated by multiple miRNAs at T2 but was only regulated by bta-miR-33a at T5 (Fig. 4A, S7B). For RE, 13 out of 16 functions were regulated by multiple miRNAs primarily bta-miR-124a, −124b (Fig. 4B, S7C). Only extracellular space and extracellular region (bta-miR-152), and negative regulation of molecular function (bta-miR-22.3p) were regulated by a single miRNA (Fig. 4B).

### Varied mucosa-attached microbiota-miRNAomes crosstalk in response to STEC O157 colonization in a stx2a-dependent manner

Next, the potential miRNAs that target DEGs were further predicted using miRWalk (Version 3) and verified using miRNAome profiles with expression levels that negatively correlated with host transcripts (Pearson’s *r* < − 0.99 and *P* < 0.01, defined as predicted miRNAs and were used for subsequent analysis).

**Fig. 5:**
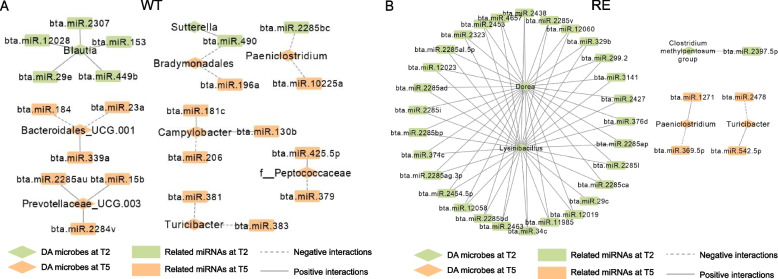
Crosstalk between differential abundant microbes and host predicted miRNAs for WT (**A**) and RE (**B**). The color of each node refers to different sample collection times (T2 or T5). The rhombus and rectangle shapes refer to microbes and miRNAs, respectively. The solid and dotted lines refer to positive and negative interactions, respectively

Relationships between predicted miRNAs and the relative abundance of genera were then evaluated. These correlations were limited to differentially abundant taxa to strengthen the potential biological relevance of miRNA–microbe interactions during colonization. The relative abundance of *Blautia* was positively related to the expression of five miRNAs (bta-miR-12028, −2307, −153, −449b, and −29e) at WT-T2 (Fig. 5A). Besides, the relative abundance of *Bradymonadales* was negatively related to the expression of bta-miR-490 at WT-T2, while negatively related to the expression of bta-miR-196a at WT-T5 (Fig. 5A). *Paeniclostridium* was the other DA bacteria that negatively related to the expression of miRNAs at both T2 (bta-miR-2285bc) and T5 (bta-miR-10225a) in WT (Fig. 5A). At WT-T5, the relative abundance of *Prevotellaceae UCG* 003 was solely positively related to the expression of miRNAs (bta-miR-2285au, −15b, and −2284v); however, *Turicibacter* exhibited only negative relationships with miRNA expressions (bta-miR-381, −383, Fig. 5A). For RE, both *Dorea* and *Lysinibacillus* were related to expressions of 29 miRNAs at T2 (Fig. 5B). At RE-T5, the relative abundance of *Paeniclostridium* and *Turicibacter* were positively associated with the expression of bta-miR-1271 and −542.5p, respectively, while negatively related to the expression of bta-miR-369.5p and −2478, respectively (Fig. 5B).

Further analysis using linear regression models revealed that both abundant and intermediate microbes were associated with log_10_ fecal O157 shedding (Fig. S8A, B). An average of 4.2 ± 1.10, 0.84 ± 1.01, 3.82 ± 1.42, and 2.68 ± 2.43 CFU/g log_10_ fecal STEC O157 were identified at WT-T2, WT-T5, RE-T2, and RE-T5, respectively. At WT-T2, the relative abundance of *Negativibacillus*, *Bradymonadales*, *Paeniclostridium*, and *UCG* 002 was negatively related to log_10_ fecal O157 shedding but a positive relationship was observed between shedding and *Treponema* (Fig. S8C)*.* Moreover, the log_10_ fecal O157 shedding level was negatively related to the expression of bta-miR-190b (*R*_adj_^2^ = 0.81, *P* = 0.009) and bta-miR-2285bt (*R*_adj_^2^ = 0.70, *P* = 0.024) at WT-T2 and was positively related to the expression of bta-miR-2285u (*R*_adj_^2^ = 0.94, *P* = 0.004) at WT-T5 and bta-miR-376e (*R*_adj_^2^ = 0.79, *P* = 0.005) at RE-T5 (Fig. S9).

### Mucosa-attached microbiota coupled with miRNAomes affected host cell types predicted by transcriptomic profiles

**Fig. 6:**
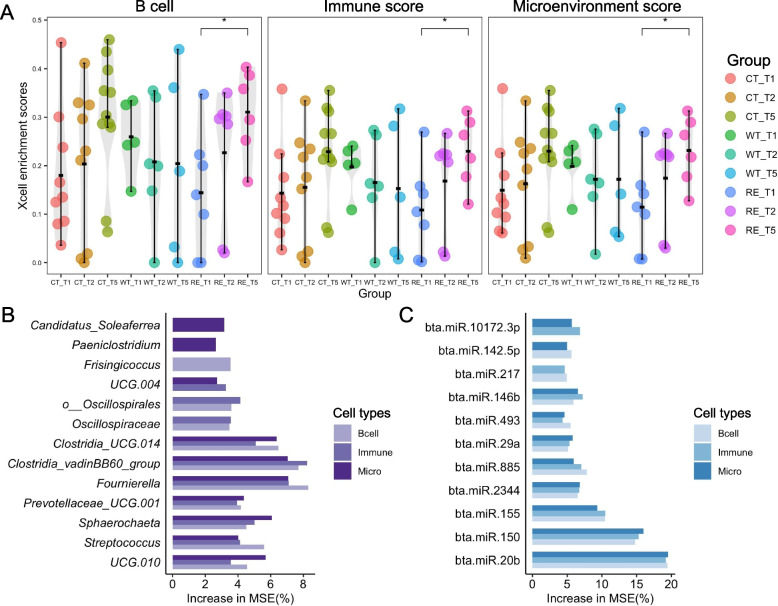
The most enriched cell types by xCell and predictive microbes and miRNAs by the random forest model **A**. The Beeswarm plot showing the xCell enrichment scores for the most enriched cell types for samples collected from calves without challenge and post-challenge for WT and RE at T2 and T5. The Immune Score is defined as the adjusted scores of B-cells, CD4+ T-cells, CD8+ T-cells, Dendritic cells, Eosinophils, Macrophages, Monocytes, Mast cells, Neutrophils, and Natural Killer cells. The Microenvironment Score is defined as the sum of the Immune Score and Stroma Score (the adjusted scores of Adipocytes, Endothelial cells, and Fibroblasts). The *P *value < 0.05 as a significance. The predictive top ten mucosa-attached microbes (**B**) and host miRNAs (**C**) related to enriched cell types using a random forest model, respectively. The contribution of microbes (**B**) and host miRNAs (**C**) to cell types was evaluated using the percent increase in mean squared errors (increased in MSE%, x-axis)

The host cell type enrichment analysis was performed using host transcriptome by “xCell” R package [26]. The host immunity, microenvironment, as well as B cells were highly enriched across T1 to T5 for CT, WT, and RE and were higher (*P* < 0.05) at RE-T5 compared to RE-T1 (Fig. S10, 6A). The random forest model revealed that the top ten microbes (Fig. 6B) and miRNAs (Fig. 6C) were related to B cells, host immunity, and microenvironment. Particularly, *Clostridia UC*G.014, *Clostridia vadinBB*60 group, *Fournierella*, *Prevolellaceae UCG.*001, *Sphaerochaeta*, *Streptococcus*, and *UCG*.010 as well as bta-miR-146b, −493,−29a,−885,−2344,−155,−150, and −20b were related to B cells, host immunity, and microenvironment simultaneously (Fig. 6B, 6C).

### MiRNAomes affected host-microbiome interactions through toll-like receptor gene expressions

**Fig. 7:**
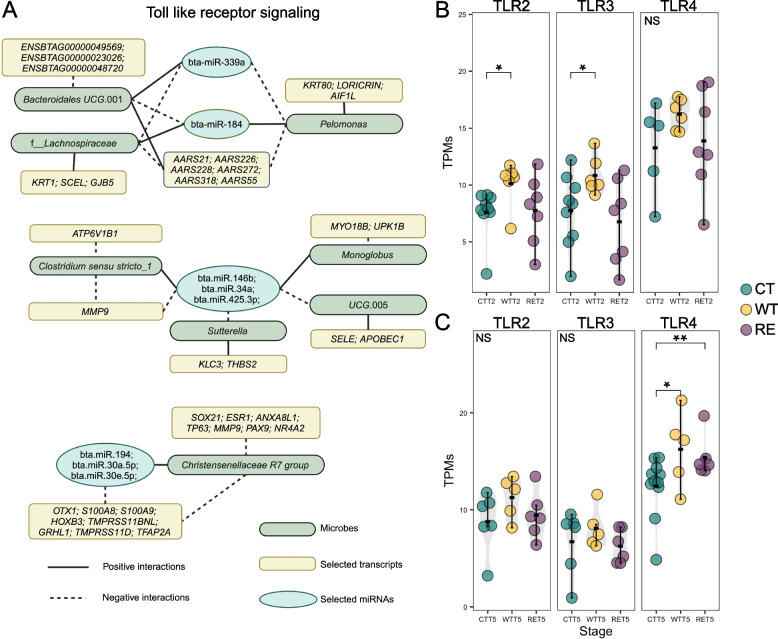
MiRNAs with functions relevant to Toll like receptor signaling mediate host-mucosa-attached microbiota interactions. The miRNAs with predicted functions associated with toll-like receptor signaling **A** for WT-T5. The expressions of *TLR2*, *TLR3*, *TLR4* at T2 (**B**) and T5 (**C**) for CT, WT, and RE

The predicted miRNAs regulating host-microbiome interactions were analyzed using Spearman correlation (absolute *R* > 0.8 and *P*_adj_ < 0.01) and such crosstalk was only observed for WT from T1 to T5 (Fig. S11, Fig. 7A). Bta-miR-1247.5p and bta-miR-490 were the only miRNAs associated with host transcriptome and microbiota at T1 and T2, respectively (Fig. S11A, B). At T5, host-microbiome interactions were regulated by miRNAs with functions relevant to the toll-like receptor (TLR) signaling pathway (Fig. 7A). Correspondingly, expressions of *TLR2* and *TLR3* were higher at WT-T2 compared to CT-T2 and *TLR3* also exhibited higher expression in WT-T2 compared to CT-T2 (Fig. 7B). At T5, the expression of *TLR4* was significantly higher at WT and RE compared to CT; however, *TLR2* and *TLR3* did not differ (Fig. 7C). Additionally, predicted miRNAs with functions relating to epithelial proliferation, mucin barrier integrity, and cell metabolism were also observed for shaping host-microbiome interactions at WT-T5 (Fig. S11C).

## Discussion

Our study comprehensively evaluated if host mechanisms including transcriptome and miRNAome could respond to strain-specific mucosa *E*. *coli* O157 colonization and revealed the mechanism of mucosa-attached microbiota conferring *E*. *coli* O157 colonization under host-microbiome interaction context in a virulence factor-dependent manner in cattle.

Although beef cattle are considered the asymptotic carrier of *E*. *coli* O157, substantial studies observed host global and local immunities were responsible for the colonization of *E*. *coli* O157 at RAJ mucosa in beef cattle [3, 7, 19, 27, 28]. In calves challenged with Stx2a-producing *E*. *coli* O157, the rectal antibody responses to *E*. *coli* O157 antigens (H7, Tir, and intimin-specific IgG1) were enhanced [29]. Subsequent evidence indicated that host immune responses (particularly transcripts enriched in B and T cell signaling pathways) to *E*. *coli* O157 colonization were affected by specific mucosa-attached bacteria [3]. However, the host responses to O157 colonization in beef cattle were not limited to immunity [19, 30, 31]. Other studies identified that molecular regulatory mechanisms regulating cholesterol metabolism and restriction of epithelial cell generation were associated with super-shedding, indicating that non-immune factors may also influence *E*. *coli* O157 colonization in beef cattle [19, 32]. The current study based on host longitudinal transcriptomic responses and active mucosal attached microbiota using the strain-specific *E*. *coli* O157 calf model revealed the arms race between *E*. *coli* O157 and the host. Based on the functional analysis and networks summarizing predicted host functions, our study observed that host responses were typically related to the extracellular structures, which is important for *E*. *coli* O157 colonization and subsequent host recognition [33, 34], and tissue integrity. Notably, such host responses were largely dependent on Stx2a. We found that colonization of wild-type could timely alter host peptidase activities at the transcriptomic level when calves become SS (T2). DEGs with functions relevant to peptidase inhibitor activity were downregulated at WT-T2, resulting in the potential increased production of peptidase in the host. Peptidases are enzymes capable of cleaving proteins and are widely distributed on cell surfaces [35]. Although enzyme activities were not measured in the study, a previous study reported that peptidases function in immune responses such as peptide‐mediated inflammatory responses and T cell activation [35], which may play a key role in the direct killing effect on WT *E*. *coli* O157 that did not produce Stx2a.

Furthermore, we speculate that Stx2a does not appear to modulate local antibody responses to STEC since host transcriptomic profiles in RE were similar to those in CT at T2. Yet, host transcriptional responses elicited by WT and RE *E*. *coli* O157 largely overlapped at T5, indicating that both strains activated similar gene expression programs in the host at T5, likely due to shared virulence factors (i.e., intimin) and conserved mechanisms of host recognition (i.e., through Toll-like receptor signaling). Interestingly, several differentially expressed genes such as *S100A9* and *IL36G* are central to innate immune regulation. *S100A9* plays a critical role in neutrophil chemotaxis, antimicrobial activity, and is a known marker of mucosal inflammation [36, 37]. *IL36G* contributes to epithelial defense and Th17-associated inflammation [38, 39]. These genes are involved in immune pathways including the IL-17 and NF-κB signaling pathways, which are known to be critical in responding to bacterial pathogens at mucosal surfaces [40, 41]. The enrichment of these and related genes suggests a coordinated mucosal immune response to *E*. *coli* O157 colonization, particularly in the context of host–pathogen interaction and virulence factor recognition. To support these transcriptomic findings and provide in-depth mechanistic understanding, future studies would benefit from including pathway enrichment analyses such as KEGG or Reactome.

What is more, mucosa-attached microbes play a key role in host responses to *E*. *coli* O157 colonization in beef cattle [4, 42, 43]. A previous study using the same cohort of calves revealed that host immune responses to *E*. *coli* O157 colonization were affected by mucosa-attached microbial community assembly and microbe-microbe interactions at RAJ, suggesting gut commensals are responsive to *E*. *coli* O157 colonization and host [3]. Our study further suggested the potential ecological mechanism of mucosa-attached microbiota enhancing *E*. *coli* O157 colonization through the shift of ecological niche. The niche is a core concept to explain the spatial and temporal dynamics of microbial communities divergent in distribution, abundance, and resource use [20, 44]. The niche breadth highlights the biotic and abiotic needs of microbial communities and is central but not limited to ecosystem functioning [45]. To date, the niche and its ecosystem interpretation in mucosa-attached microbiota under the context of pathogen colonization in the gut has not been widely studied. Our study revealed a decrease in niche breadth of mucosa-attached microbiota post-challenge in both WT and RE, indicating a potential selective pressure exerted by *E*. *coli* O157 colonization that was not subjected to Stx2a. Such competition between *E*. *coli* O157 and mucosa-attached microbiota may lead to a more specialized microbial community as the previous study reported [3]. Correspondingly, functionality relevant to microbial metabolism including fermentation and anaerobic chemoheterotrophy (Energy-yielding) was significantly decreased post-challenge, providing the knowledge of how mucosa-attached microbiota respond to *E*. *coli* O157 colonization. While functional predictions from 16S rRNA gene sequencing provide useful insights, they are inherently limited by database accuracy and taxonomic resolution. However, given the high proportion of host DNA in mucosa-attached microbiome samples and the lack of effective methods to separate host and microbial DNA, generating metagenomic datasets would require extremely deep sequencing, increasing costs and potential biases. Therefore, we employed 16S rRNA gene amplicon sequencing for microbial profiling and used RNA-based analyses as a complementary approach.

Microbial abundance is an important component of the niche concept and most previous studies focused on rare and abundant bacteria and their effects on microbial communities and ignored intermediate bacteria and their contributions to microbial community structure and function largely ignored [46, 47]. In our study, intermediate bacteria contributed more to the niche occupancy and functionality, highlighting the role of intermediate bacteria in driving mucosa-attached microbiota and interactions with *E*. *coli* O157 colonization. Besides, the decrease in ecological niche diversity during the pathogen colonization was observed for both WT and RE independent of Stx2a production, suggesting niche limitation of mucosa-attached microbiota to pathogen colonization is not subject to pathogen virulence factors.

Furthermore, our study is the first to demonstrate that miRNAomes can influence host mechanisms during *E*. *coli* O157 colonization, with miRNA-mediated host-microbiota interactions modulating host responses in a Stx2a-dependent manner. The previous study revealed that tissue-associated miRNAs were differentially expressed in SS compared to NS in beef cattle [16]. Our study further revealed that differential expressions of miRNAs were more sensitive to the production of Stx2a. Zooming into patterns of miRNAome regulating host functions, it is the first time revealed the regulatory patterns of miRNAs to host functions were different possibly due to the production of bacterial virulent factor (Stx2a). The aforementioned host GO terms enriched by DEGs (i.e., extracellular region, and serine-type endopeptidase inhibitor activity) were consistently regulated by multiple miRNAs such as bta-let-7i and bta-miR-181b. The bta-let-7i, a member of the let-7 family is a downstream effector of toll-like receptor (TLR) signaling [48, 49] and the recognition of bacterial lipopolysaccharides (LPS) in Gram-negative bacteria (i.e., *E*. *coli*) to TLR is capable of inducing host immune responses [50]. The increased expression of *TLR2* in response to high fecal shedding of O157 (T2) in WT calves supports the idea that pathogen-associated molecular patterns (PAMPs) from *E*. *coli* O157 are detected by the host, triggering immune responses [51, 52]. And subsequent activation of host immune cells promotes the secretion of inflammatory cytokines such as IL-17 [53, 54] and can be regulated by miRNAs (i.e., bta-miR-181b) [55]. However, the differential regulation of *TLR2* in RE, where Stx2a did not affect *TLR2* expressions, suggests that Stx2a might actively inhibit *TLR2*-mediated immune recognition, potentially allowing the pathogen to evade proper immune detection in the mucosal environment.

Interestingly, *TLR3*, which senses viral double-stranded RNA and is highly expressed in innate immune cells for virus-mediate immunities [56], was highly expressed in WT-T2. A previous study revealed that *TLR3* agonists can increase intracellular killing of pathogenic encapsulated *E*. *coli* K1 [57]. If the *TLR3* activation and virus-mediate responses coupled with host-microbiota interactions could shape host reactions toward *E*. *coli* O157 warrants further exploration. Therefore, the cooperativity of multiple miRNAs at WT-T2 could potentially enable hosts to recognize the pathogen through the activation of TLR and highlight their potential as key regulators in host–pathogen interactions, possibly contributing to host resilience or susceptibility to specific *E*. *coli* O157 strains.

While our integrative multi-omics approach provides valuable insight into miRNA-mRNA-microbiota interactions during *E*. *coli* O157 colonization, it is important to acknowledge that these relationships remain correlative. Although our data suggest that miRNAs such as bta-miR-181b may influence TLR signaling pathways and microbial composition, further experimental validation is required to establish causality. Functional assays—such as miRNA overexpression or knockdown in bovine-derived intestinal organoids or epithelial cell lines, as well as dual-luciferase reporter assays to confirm direct binding between specific miRNAs and their predicted mRNA targets (e.g., bta-miR-181b and TLR4)—will be essential for confirming the regulatory roles proposed here. However, the generation and maintenance of appropriate in vitro and/or ex vivo models for bovine tissues, along with the development of luciferase reporter constructs with cloned 3′UTRs of specific bovine genes, require substantial time and infrastructure investment. Given these constraints, our study focused on high-throughput sequencing and integrative bioinformatics to identify candidate miRNA–mRNA interactions and their potential links to microbial community dynamics. We view these computational predictions as a valuable first step and fully support follow-up studies using functional assays to validate and extend our findings.

Moreover, we observed the remarkable multiplying effect (defined as one miRNA can regulate multiple host functions in our study) of bta-miR-33a (WT-T5) and miR-124a, b (RE-T5) regulating host functions. The bta-miR-33a and miR-124 family affect host lipid and fatty acid metabolism, and such metabolism plays a major role in regulating host immunity [58–62]. Hence, the multiplying effect of these miRNAs mediating host responses to *E*. *coli* O157 indicates a dual role of miRNAs in metabolic regulation and immune modulation during *E*. *coli* O157 colonization. The altered lipid metabolism has been reported in SS, and fatty acids and other metabolites are central to both immune responses and metabolic regulation [32]. These indicate that their variation during colonization could provide deeper insights into how the host adapts to *E*. *coli* O157. Although biopsied samples were not enough for further metabolomics, it is needed to evaluate fatty acid changes and other metabolite variations in the future. In addition to miRNA-regulated host gene expressions, host gene expressions can be regulated by other mechanisms such as alternative splicing, and epigenetics [63, 64]. If and how other mechanisms constitute the cause-and-effect responses to O157 colonization warrants further research.

What is more, it has been speculated that host miRNAs can be secreted by extracellular vesicles to the mucosal layer and promote interactions with microbiota [65–67]. Yet, the direct evidence of interplay between mucosa-attached microbiota and miRNAomes is still lacking. Based on our integrated analysis, we speculated that host miRNAs could be mediators for varied host-microbiome interactions for strain-specific *E*. *coli* O157 colonization. For instance, our previous study revealed that the opportunistic pathogenic *Paeniclostridium* mediated interactions between host immune-related pathways and *E*. *coli* O157 colonization [3]. *Paeniclostridium* was directly associated with expressions of bta-miR-2285bc (T2) and bta-miR-10225a (T5) at WT, bta-miR-1271 and bta-miR-359.5p (RE-T2), indicating previously reported mucosa-attached microbiota affecting host functions are mediated by host miRNAs. In particular, miRNAs such as bta-miR-146a, −339a, and −194 associated with mucosa-attached microbes and transcripts at WT-T5 can be induced by *TLR4*. The miR-146a can regulate the crosstalk between intestinal epithelium, microbial components, and inflammatory stimuli [68, 69]. Microbial interactions with miR-146a were specific to WT-T5 and functions of *Sutterella* and *Monoglubus* were related to mucin, which constitutes an important trait shaping mucosa-attached microbiota compositions [70, 71]. The bta-miR-194 was linked to the *Christensenellaceae* R7 group, which functions in short-chain acid production and is beneficial to host homeostasis [72]. Therefore, we highlight the TLR subtype activation dynamics to *E*. *coli* O157 could result in various microbiota-host interactions in response to O157 colonization and the stx2a production. The presence of miRNA-mediated crosstalk in cattle extends this concept beyond mouse models [18] and highlights the potential for miRNAs as key mediators of host-microbiome interactions in different species. Also, miRNAomes could be affected by not only commensals, but also affected by pathogen challenge and Stx2a. Compared to the study that only highlights either miRNAomes or commensals affecting super-shedding and O157 colonization [10, 14, 16], our findings provide novel insights into the mechanisms of O157 colonization and super-shedding through the coregulation of miRNAomes and mucosa-attached microbiota.

**Fig. 8:**
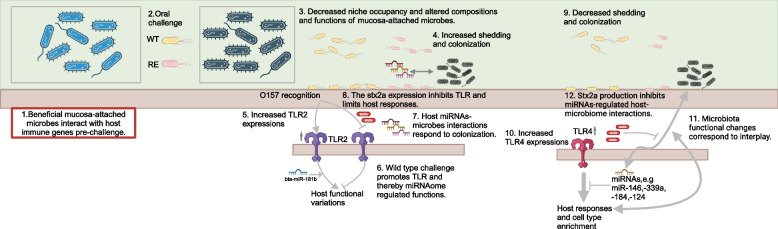
The schematic diagram of host mechanisms of *E*. *coli* O157 colonization regulated by mucosa-attached microbiota and miRNAome. Step 1 in the box was based on the previous findings using the same cohort of calves. Steps 2–4: The oral challenge of WT and RE strains could decrease niche occupancy of mucosa-attached microbiota and its functionality and such changes in mucosa-attached microbiota could contribute to O157 shedding and mucosa colonization. Step 5: The mucosa O157 colonization altered Toll-like receptor 2 (*TLR2*) gene expressions. Step 6: In the absence of Stx2a production in WT, the expression of *TLR2* promoted bta-miR-7i mediated host functions in response to O157 colonization. Step 7: Host miRNAs could interact with mucosa-attached microbes as the result of O157 colonization. Step 8: The production of Stx2a may inhibit host *TLR2* expression and block host recognition of RE O157 strain at T2. Step 9: At T5, the O157 fecal shedding was decreased. Step 10: The expression of *TLR4* was increased regardless of the production of Stx2a, resulting into TLR4- and host miRNAs-mediated host responses. Steps 11–12: The mucosa-attached microbiota could interplay with host miRNAs and host transcriptomes in response to WT O157 colonization, yet the production of Stx2a may block miRNAs-regulated host-microbiome interactions

Combining the findings above, we proposed the schematic pathogen-microbiota-host axis with/without miRNAome regulation (Fig. 8). The decreased niche occupancy by mucosa-attached microbiota due to the challenge of strain-specific O157 could be the prerequisite for varied host-microbiota interactions. The *TLR* subtype expression shifts may lead to varied miRNAome- and mucosa-attached microbiota-mediated pathogen-host interplays. The production of Stxa2 could limit host recognizing O157 or block miRNAome-mucosa-attached microbiota crosstalk. This proposed schematic model is useful to improve our understanding of how *E*. *coli* O157 confers mucosa-attached microbiota for colonization and host mechanisms regulating microbiota-pathogen interactions, particularly in the context of Stx2a production, providing novel knowledge for integrated multi-omics study to explore the pathogen colonization ecology. Future studies are required to verify if the shift of TLR subtypes is related to other critical bacterial virulence factors (i.e., type III secretion system) and long-term evaluation of miRNAs regulated host-microbiota interactions contributing to *E*. *coli* O157 colonization.

## Conclusion

Our comprehensive multi-omics assessment revealed host mechanisms in response to the O157 colonization and the ecological influence of mucosa-attached microbiota in affecting *E*. *coli* O157 colonization. Furthermore, host responses to *E*.* coli* O157 colonization are influenced by the pathogen’s virulence factor, particularly Stx2a, which alters host immunities. Stx2a impacts the interactions between the host (via miRNAome and transcriptome) and mucosa-attached microbiota through various mechanisms, including shifts in *TLR* subtype expression across different colonization stages. However, our assumptions warrant further in vitro on-a-chip models to verify such host-microbiome interactions. Overall, our findings offer novel insights into the joint effect of mucosa-attached microbiota and miRNAomes on host–pathogen interactions, enhancing our understanding of how miRNAomes and mucosa-attached microbiota contribute to maintaining host homeostasis.

## Materials and methods

### Animal study and sample collection

All animal work was carried out at the Moredun Research Institute (MRI) under Home Office License 70/7914 granted by the UK Home Office under the Animal (Scientific Procedures) Act 1986 and was approved by the MRI animal care and ethics review committee.

The wild-type O157 strain (WT 21/28^*stx2−stx2c*+^) was subjected to genetic removal of insertion sequence ISEc8 within the coding region of *stx2a* gene subunit A to generate the lab-reconstructed O157 strain (RE 21/28^*stx2a*+*stx2c*+^) [19, 29]. The removal of such insertion seq ISEc8 is to generate functional stx2a in lab constructed O157 strain. And the deletion of ISEc8 was confirmed by PCR and sequencing by Fitzgerald et al. [19]. Due to biosafety requirements and the need for controlled infection conditions, seven calves were selected for each challenge group (WT and RE) and matched numbers of calves in the control group (*n* = 7 for each group, *n* = 14 for total control calves). One calf in WT and three claves in CT died before starting the trial. Therefore, there were 6 calves in WT, 7 calves in RE, and 11 calves in CT for the trial (WT, *n* = 6; RE, *n* = 7; CT, *n* = 11). Fecal samples of all 24 calves (WT, *n* = 6; RE, *n* = 7; control, *n* = 11) were pre-screened five times per week using immunomagnetic separation per manufacturer’s instructions (Dynabeads anti-STEC O157; 75 Invitrogen, Paisley, UK) to ensure negative STEC O157 prior to the oral challenge. Also, the fecal culture and reverse transcription quantitative PCR confirmed calves to be STEC O157, stx1, and stx2 negative before entering the trial. All calves were of the same breed and age, and weaned at the same time and fed hay and calf concentrate through the trial and each challenge group of calves was allocated to different rooms at the MRI high security unit (HSU) except control calves which were conventionally on the MRI farm. Although housing location differed due to biosafety constraints, every effort was made to standardize environmental exposures across groups. Claves from WT and RE were orally challenged by orogastric intubation with ~ 10^9^ CFU of each strain in 10 mL of lysogeny broth (for CT, an equal amount of broth with saline was used). The RAJ mucosa samples were collected using a mild-evasive biopsy approach from CT, WT, and RE at 3 days before challenge (D-3, T1), 7 days (D7, T2), and 26 days post-challenge (D26, T5) and were stored at − 80 °C. To minimize luminal contamination, visible digesta and loosely adherent material were gently removed with sterile PBS rinses prior to sample preservation. Details of the fecal sample collection procedure and fecal O157 counts were described in our previous study [19, 29].

### RNA extraction, host RNA, and miRNA sequencing

The fine-grounded tissue samples were subjected to RNA extraction. For RNA sequencing, RNA was isolated from 0.1 g tissue using TRIzol reagent (Invitrogen Corporation, Carlsbad, CA, USA), and was purified using the RNeasy MinElute Cleanup kit (QIAGEN, Valencia, CA, USA). The quality of extracted RNA was assessed individually using Agilent 2200 TapeStation (Agilent Technologies, Santa Clara, CA, USA) and Qubit 3.0 Fluorometer (Invitrogen, Carlsbad, CA, USA). Only RNA with an integrity number (RIN) greater than 7.0 and a ratio of A260/A280 from 1.7 to 2.4 was used for subsequent RNA/miRNA seq library construction.

For RNA sequencing, extracted total RNA (1 μg) was used for library construction using the Truseq Stranded Total RNA Sample Preparation kit (Illumina, San Diego, CA, USA) following the manufacturer’s manual. For miRNA sequencing, 1 µg of total RNA was used for library preparation using a QIAseq miRNA Library Kit (QIAGEN, Valencia, CA, USA) following the manufacturer’s instructions. The RNA and miRNA sequencing were performed at the Genome Quebec Innovation Centre (Montreal, PQ, Canada) using paired-end (100 bp) sequencing on an Illumina NovaSeq 6000 system (Illumina, San Diego, CA, USA) and single-end (100 bp) sequencing on an Illumina NovaSeq 6000 S1 system (Illumina, San Diego, CA, USA), respectively.

### Host transcriptome and miRNAome data processing

Host RNA sequencing reads were first subjected to the quality filter and adapter trimming using FastQC and bbDuk. The filter high-quality reads were then mapped against the reference bovine reference assembly ARS-UCD 1.2.99 using STAR (Version 2.7.1a). Feature counts that count mapped reads for exons were then generated using subread (Version 2.0.0) and normalized into TPM (transcripts per million) using the formula: (exon reads in genes)/(total exon reads) × 1 million. Transcripts with an average TPM > 0.2 were considered as detected transcripts that were used for further analysis [73]. The miRNA profiles were analyzed using miRDeep2, by which the 3′ adapter sequence was clipped, and reads shorter than 17 nts were discarded. The filtered reads were then mapped to the bovine ARS-UCD 1.2 database. The read number of detected miRNAs was normalized as counts per million (CPM), and the miRNAs with CPM > 1 were defined as expressed miRNAs.

### Host transcriptomic and miRNAomic profiles in response to STEC O157 colonization

The differentially expressed genes (DEGs) and differentially expressed (DE) miRNAs were identified using pairwise comparisons: WT vs. CT, RE vs. CT, and WT vs. RE post-challenge (T2 and T5) using the DeSeq2 package in R, respectively. DeSeq2 employs a statistical framework based on the negative binomial distribution and incorporates internal normalization and variance estimation to account for group imbalances. The log_2_ fold change of each DEG/DE miRNA was calculated using the equation: for comparisons between WT vs. CT or RE vs. CT: log_2_ fold change = log_2_ (average normalized counts in challenged calves/average normalized counts in the control group); for the comparison between WT vs. RE: log_2_ fold change = log_2_(average normalized counts in WT/average normalized counts in RE). Here, normalized counts refer to TPM and CPM for DEGs and DE miRNAs identification, respectively. The DEGs were defined as transcripts with Benjamini–Hochberg adjusted *P* < 0.05 and absolute log_2_ fold change > 1.5. The log_2_ fold change for DEGs was set to < − 1.5 or > 1.5 with negative indicating downregulations and positive indicating upregulations. The functions of DEGs were enriched using the Gene Ontology (GO, *P*_adj_ < 0.05 as the cut-off). The DE miRNAs were defined as miRNAs with Benjamini–Hochberg adjusted *P* < 0.05 and absolute log_2_ fold change > 1.5.

### Mucosa-attached bacterial 16S rRNA gene amplicon sequencing and analysis

The RNA extraction procedure and library construction for bacterial 16S rRNA gene amplicon sequencing were described in our previous study [3]. The demultiplex, quality control, denoising, removal of chimeric sequences, and generation of amplicon sequencing variants (ASVs) were performed using the QIIME2 [74]. Taxonomic classification was performed in QIIME2 using a taxonomic classifier with the SILVA database (Version 138) [74]. Only identified genera with a relative abundance > 0.01% and presented in at least half samples were included in further analysis. The Good’s coverage index > 99% indicates the proper adequacy of sequencing depth to generate bacterial profiles in each sample.

The niche breadth index can be measured by ${B}_{j}=\frac{1}{{\sum }_{i=1}^{N}{P}_{ij}^{2}}$ and was used to quantify total and abundant-specific niche occupancy of mucosa-attached microbiota post challenge for WT and RE (T2 and T5). Here, ${B}_{j}$ stands for niche breadth, ${P}_{ij}$ refers to the proportion of any genera $i$ in a given sample, $j$ and $N$ is the total number of samples [75]. The differential abundant (DA) bacteria were identified using the Limma package in R in comparison of WT vs. CT, RE vs. CT at T2 and T5, respectively (*P*_FDR-adjusted_ < 0.05). Limma with voom transformation adjusts for differences in sequencing library size and sample variability for imbalanced designs. The FAPROTAX package in R was used to map mucosa-attached microbial genera to established metabolic or other ecologically relevant functions to reveal the functional variations from pre- to post-challenge for each group.

### Relationships between miRNAs and mucosa-attached microbiota and fecal O157 shedding level

For microbiota–miRNA interaction analysis, we focused on bacterial genera that were identified as differentially abundant (DA) taxa. These genera were considered likely candidates for host-microbiota interactions related to O157 colonization or Stx2a activity. Relationships between the relative abundance of DA genera and the expression of predicted miRNAs were evaluated using Spearman rank correlation (absolute *R* > 0.8 and *P*_adj_ < 0.01). The linear regression models were adopted to reveal if the average relative abundance of abundant-specific mucosa-attached bacteria and average CPM of predicted miRNAs relate to log_10_ O157 fecal shedding levels post-challenge for WT and RE. Specifically, relationships between the relative abundance of genera and log_10_ fecal shedding levels were assessed by Spearman rank correlations (absolute *R* > 0.8 and *P*_adj_ < 0.01).

### Host cell type variations and associations with miRNAs and mucosa-attached microbiota under O157 colonization

The xCell package in R provides the most accurate and sensitive way to identify the enrichment of cell types [26] was adopted to uncover host cell type enrichments and variations in response to O157 colonization and stx2a expressions using host transcriptomic data for each group. The predictiveness of mucosa-attached microbiota and host miRNAs to enriched host cell types were evaluated using a random forest model in R (“randomForest” package). Only the top ten genera and miRNAs with the highest percent increase in mean squared error (Increase in MSE %) were considered as potential markers for host cell types.

### Longitudinal evaluations of miRNA-mRNA, miRNA-microbiota interplays, and the coregulation of miRNA and microbiota under host–pathogen interactions

The patterns of miRNA regulating DEGs were identified by Pearson correlations (*R* < − 0.99 and *P*_adj_ < 0.01) and enriched functions by DEGs among significant correlations were defined as potential functions regulated by host miRNAs. Furthermore, to understand how host miRNAs regulating mucosa-attached microbiota under host–pathogen interaction context and due to the limited DE miRNAs identified, we predicted potential miRNAs that can regulate DEGs using miRWalk (Version 3) [76] and only those existed in miRNAome profiles with expression levels significantly negatively correlated with host transcripts (defined as predicted miRNAs, Pearson’s *r* < − 0.99 and *P* < 0.01) were included for miRNA-microbiome interaction analysis. miRWalk offers a comprehensive and flexible approach to miRNA-target prediction by integrating multiple established algorithms (including TargetScan and miRanda) and databases of experimentally validated interactions (e.g., miRTarBase, TarBase) [76]. The Spearman rank correlation was adopted to identify interactions between the relative abundance of DA microbes and CPM of predicted miRNAs (absolute *R* > 0.8 and *P*_adj_ < 0.01) post-challenge for WT and RE. At last, the predicted miRNA-transcripts pairs and the relative abundance of mucosa-attached genera were further subjected to Spearman correlation to uncover the coregulation of miRNAs and microbial genera relevant to host responses of O157 colonization (absolute *R* > 0.8 and *P*_adj_ < 0.01).

## Supplementary Information

Supplementary Material 1: The heatmap showing the percentage of ASVs involved in each bacterial function for mucosa-attached microbial communities collected from calves without challenge or post-challenge for WT and RE at T2 or T5.

Supplementary Material 2: Mucosa-attached microbes divergent in abundance contribute to altered niche occupancy and altered bacterial functions. A. The Beeswarm plot showing absolute niche breadth value for abundant (left panel), intermediate (middle panel), and rare (right panel) microbes (P > 0.01 ***,0.01 ≤ P ≤ 0.05 **, 0.05 ≤ P < 0.1 *). B. The stack plot showing relative niche occupancy for mucosa-attached microbes from calves without challenge or post challenge at T2 or T5 for both WT and RE. The relative niche occupancy is defined as the sum of absolute niche breadth value for abundant-specific microbes/the sum of niche breadth value for all microbes for each group. C. The stack plot showing the contributions of abundant/intermediate/rare microbes to the most enriched bacterial functions post challenge. D. The Venn plot showing shared and specific intermediate microbes contributing the most enriched bacterial function at WT-T2 and WT-T5. E. The Venn plot showing shared and specific intermediate microbes contributing the most enriched bacterial function at RE-T2 and RE-T5.

Supplementary Material 3: Differential abundant microbes identified for WT-T2 vs CT-T2 (A), WT-T5 vs. CT-T5 (B), CT-T2 vs. RET2 (C), and CT-T5 vs. RE-T5 (D). The dashed rectangle represents the intermediate microbes.

Supplementary Material 4: The quantity of identified transcripts for CT, WT, and RE from T1 to T5.

Supplementary Material 5: The network showing relationships between enriched GO terms using DEGs at WT-T2 (A) and WT-T5 (B). Two pathways (nodes) are connected if they share 20% or more genes. Darker nodes are more significantly enriched gene sets. Bigger nodes represent larger gene sets. Thicker edges represent more overlapped genes.

Supplementary Material 6: The Sankey plot showing relationships between predicted miRNAs and functions enriched by differentially expressed genes at WT-T2 (A), WT-T5 (B), and RE-T2 (C)

Supplementary Material 7: The interaction network between the most connected predicted miRNAs (Core miRNAs) and functions enriched by differentially expressed genes (Core GO functions) at WT-T2 (A), WT-T5 (B), RE-T5 (C)

Supplementary Material 8: Relationships between mucosa-attached microbes and log10 fecal shedding level. The linear regression plot showing relationships between log10 fecal shedding level and niche breadth values occupied by abundant (A) and intermediate microbes (B). The identified significant Spearman correlations between differential abundant microbes and log10 fecal shedding level at WT-T2 (C). The log‐‐ CFU values used were the group averages for WT-T2, WT-T5, RE-T2, and RE-T5.

Supplementary Material 9: The linear regression plot showing significant relationships between the CPM of miRNAs and log10 fecal shedding level.

Supplementary Material 10: The heatmap showing the xCell enrichment value for CT, WT, and RE from T1 to T5.

Supplementary Material 11. The host miRNAs regulated host-microbiome interactions for WT-T1 (A), WT-T2 (B), and WT-T5 (C). The oval, rod, and rectangle shapes refer to miRNAs, microbes, and transcripts, respectively. The solid and dotted lines refer to positive and negative interactions, respectively.

Supplementary Material 12.

## Data Availability

All sequence data have been deposited to NCBI Sequence Read Archive (SRA) under accession numbers PRJNA991158 (RNA sequencing), PRJNA988112 (Amplicon sequencing), PRJNA1145457 (miRNA sequencing).
