# Supplementary figures and images for: Host miRNAs regulate Escherichia coli O157 mucosal colonization through host-mucosa-attached microbiota interactions in calves
Source: Microbiome. 2025 Oct 23;13:213. doi: 10.1186/s40168-025-02184-w (PMC12548129; doi:10.1186/s40168-025-02184-w)

Heatmap Plot

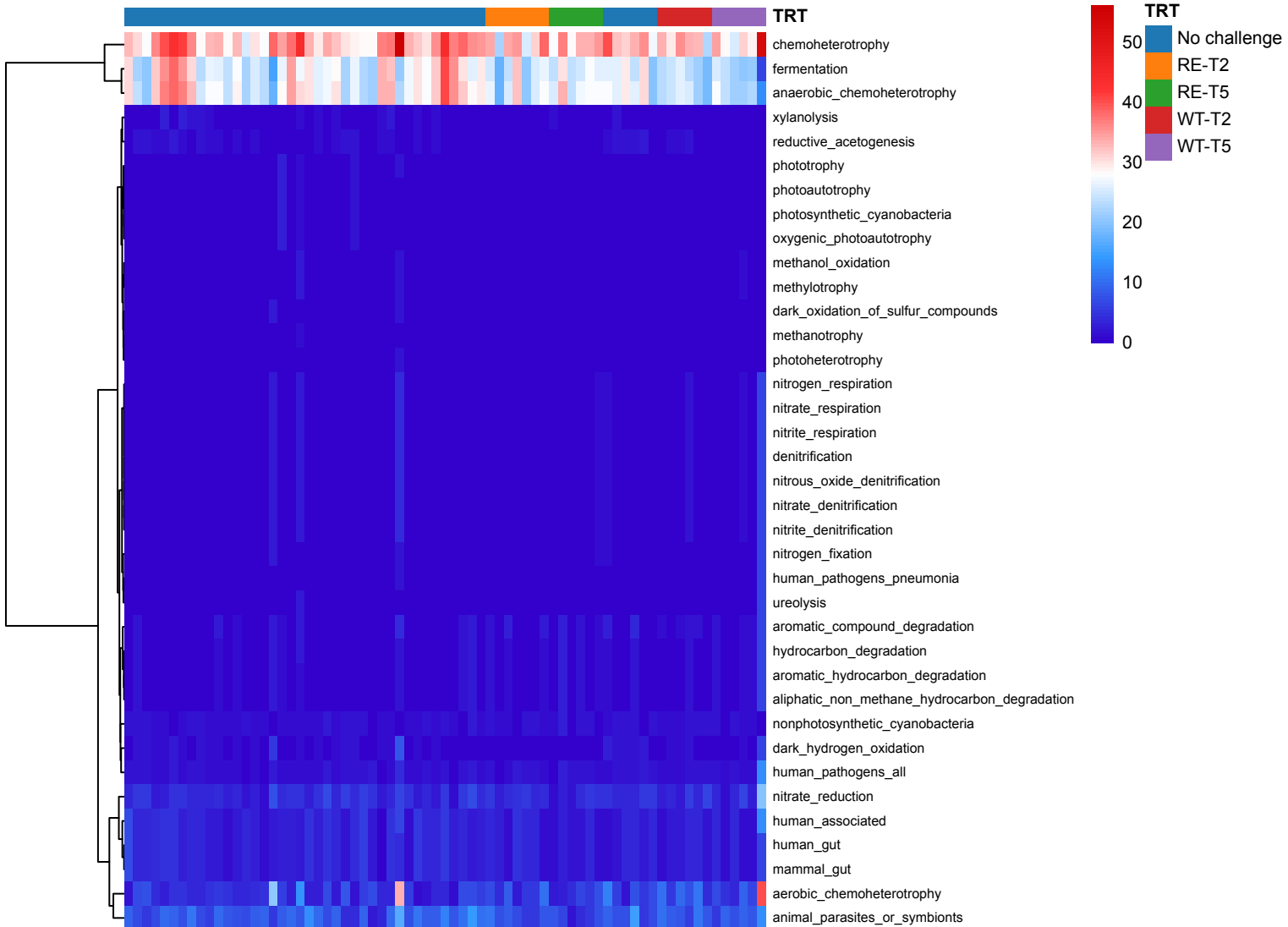

Supplement: Supplementary file 2 — Supplementary Material 1: The heatmap showing the percentage of ASVs involved in each bacterial function for mucosa-attached microbial communities collected from calves without challenge or post-challenge for WT and RE at T2 or T5. [file 40168_2025_2184_MOESM1_ESM.pdf]

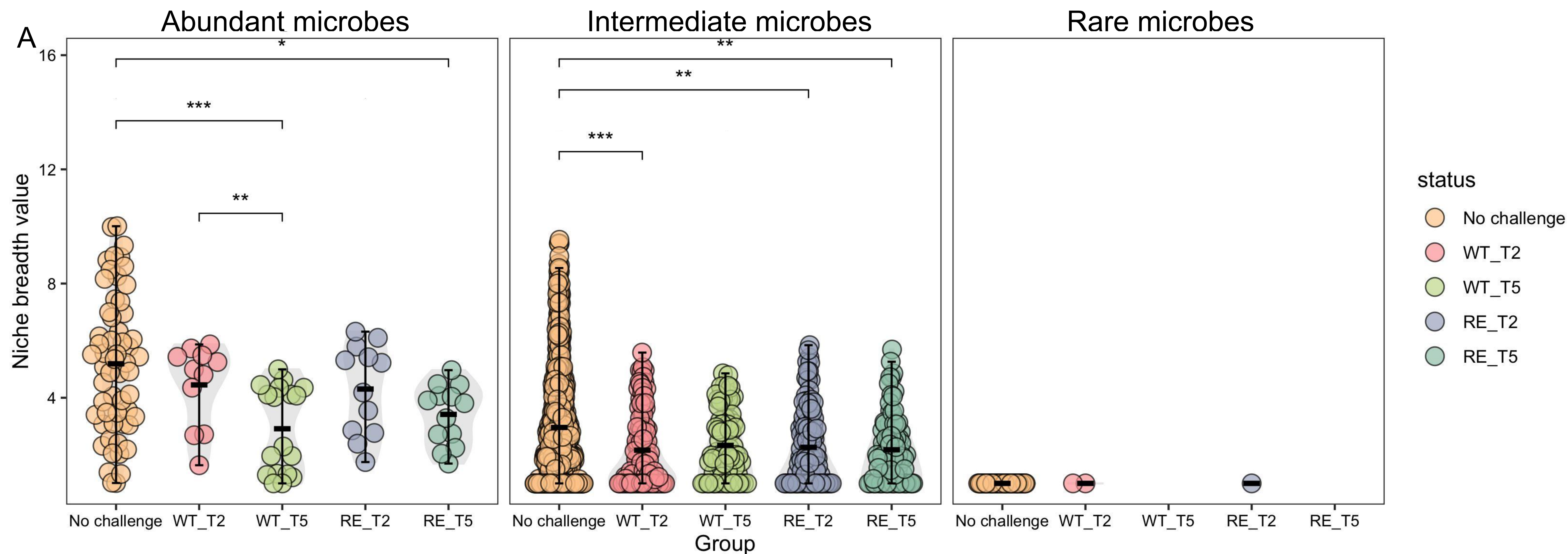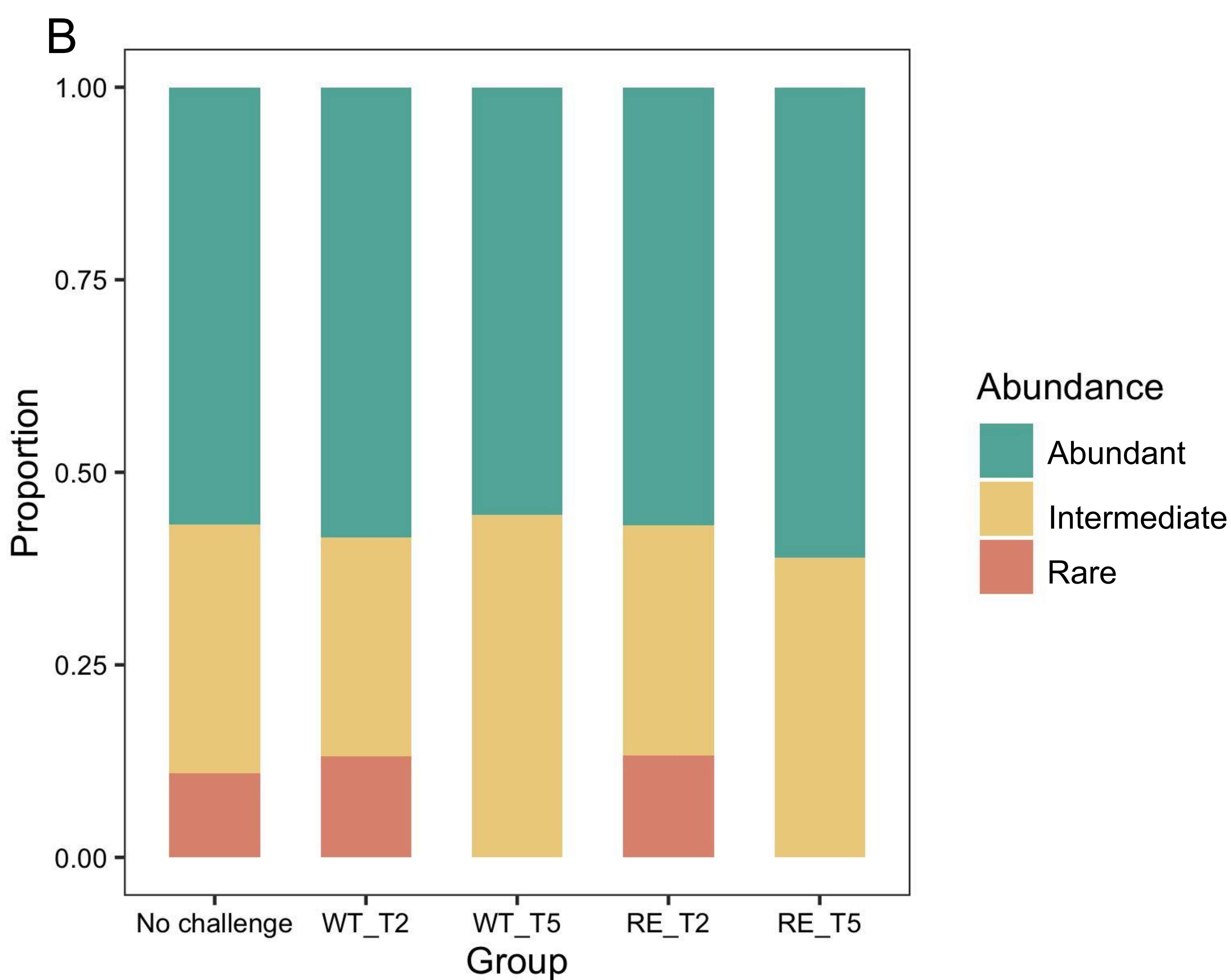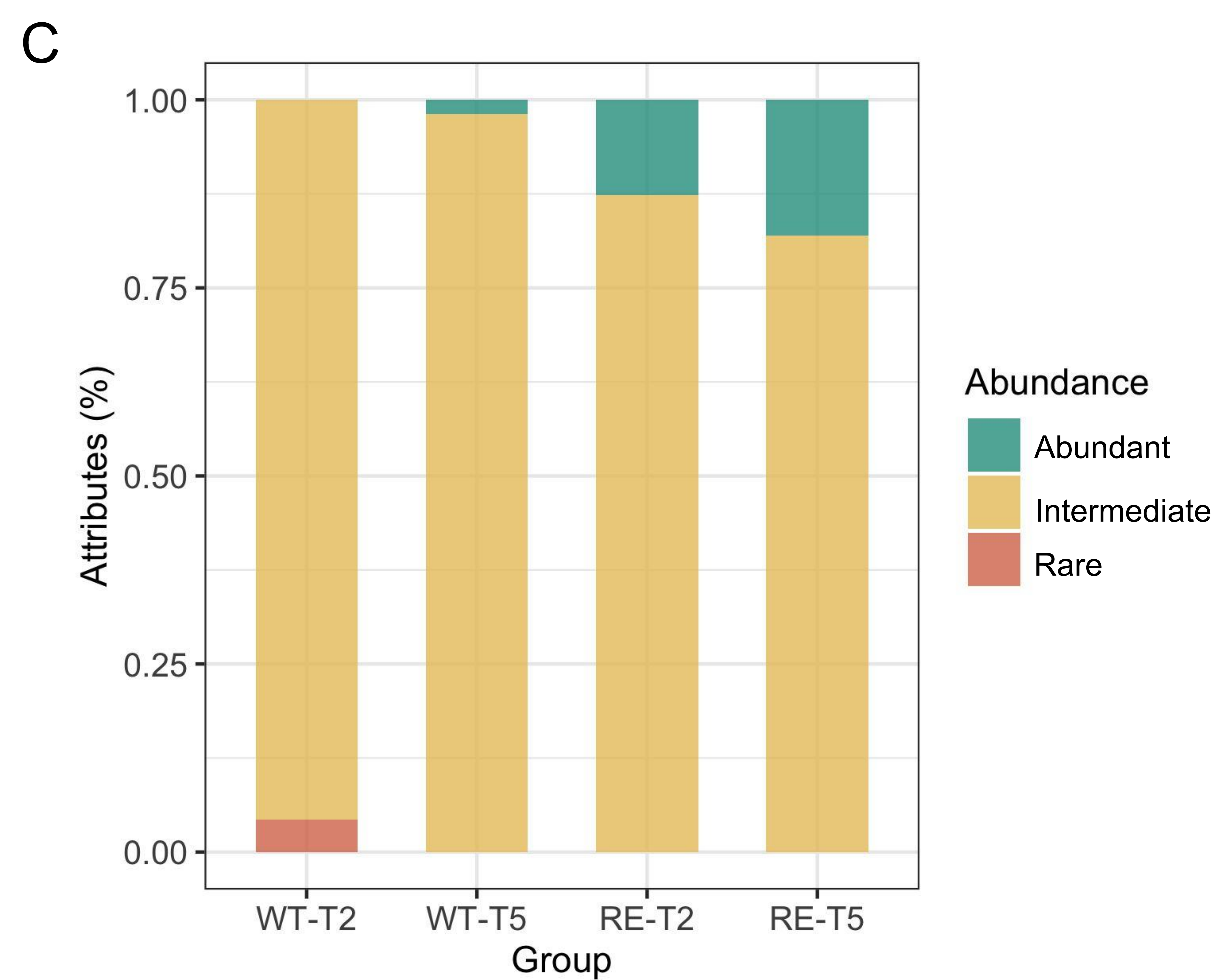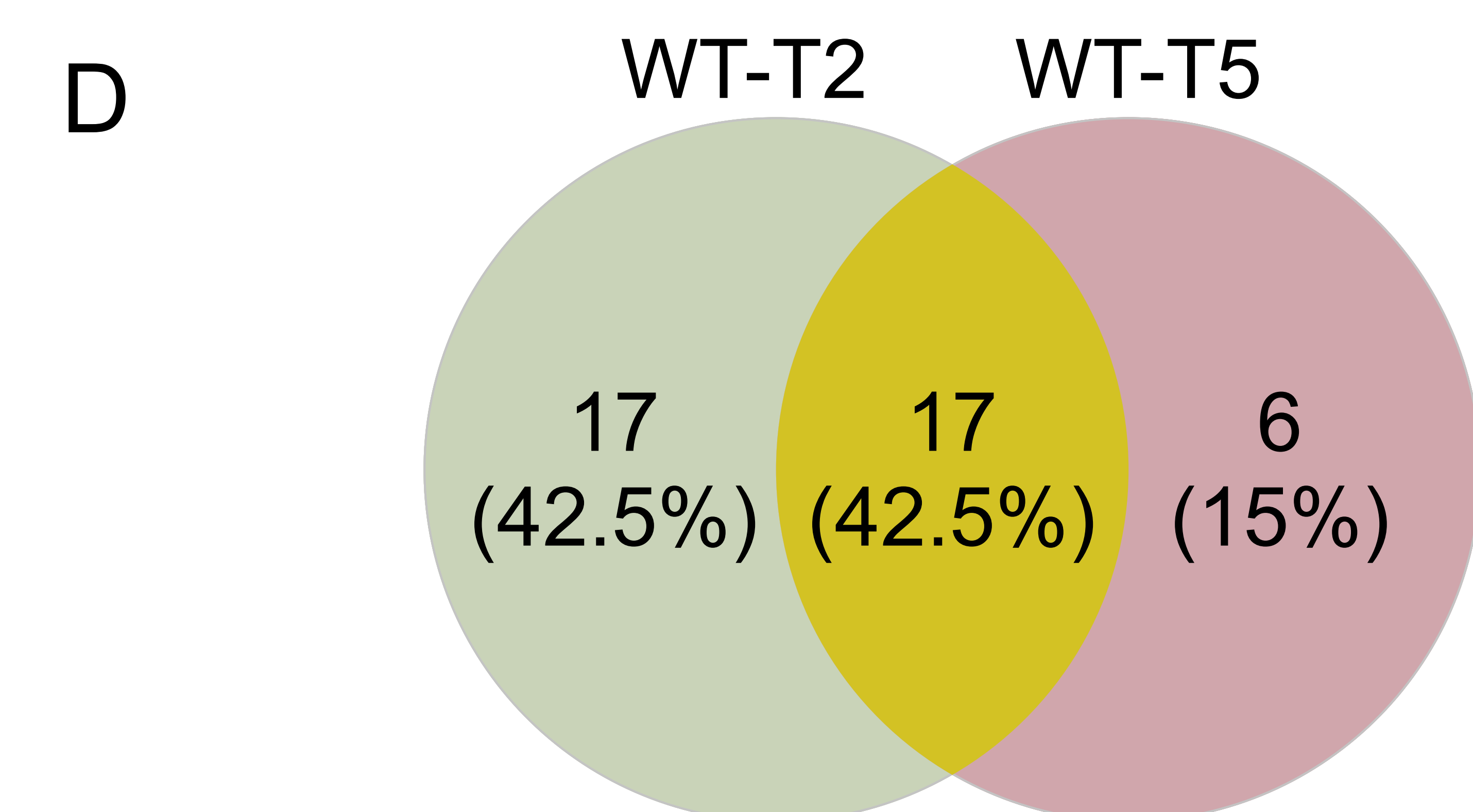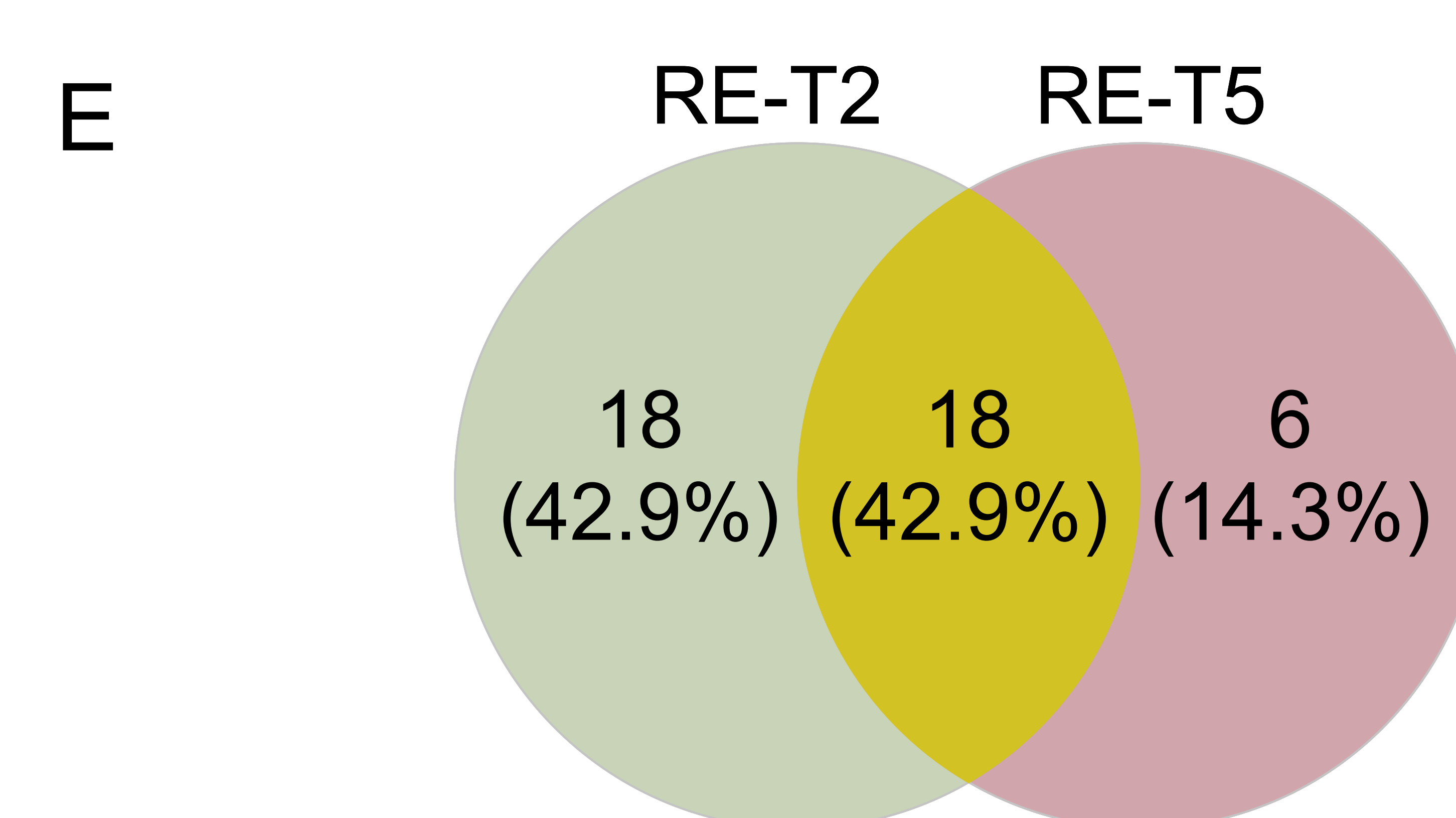

Supplement: Supplementary file 3 — Supplementary Material 2: Mucosa-attached microbes divergent in abundance contribute to altered niche occupancy and altered bacterial functions. A. The Beeswarm plot showing absolute niche breadth value for abundant (left panel), intermediate (middle panel), and rare (right panel) microbes (P > 0.01 ***,0.01 ≤ P ≤ 0.05 **, 0.05 ≤ P < 0.1 *). B. The stack plot showing relative niche occupancy for mucosa-attached microbes from calves without challenge or post challenge at T2 or T5 for both WT and RE. The relative niche occupancy is defined as the sum of absolute niche breadth value for abundant-specific microbes/the sum of niche breadth value for all microbes for each group. C. The stack plot showing the contributions of abundant/intermediate/rare microbes to the most enriched bacterial functions post challenge. D. The Venn plot showing shared and specific intermediate microbes contributing the most enriched bacterial function at WT-T2 and WT-T5. E. The Venn plot showing shared and specific intermediate microbes contributing the most enriched bacterial function at RE-T2 and RE-T5. [file 40168_2025_2184_MOESM2_ESM.pdf]

A

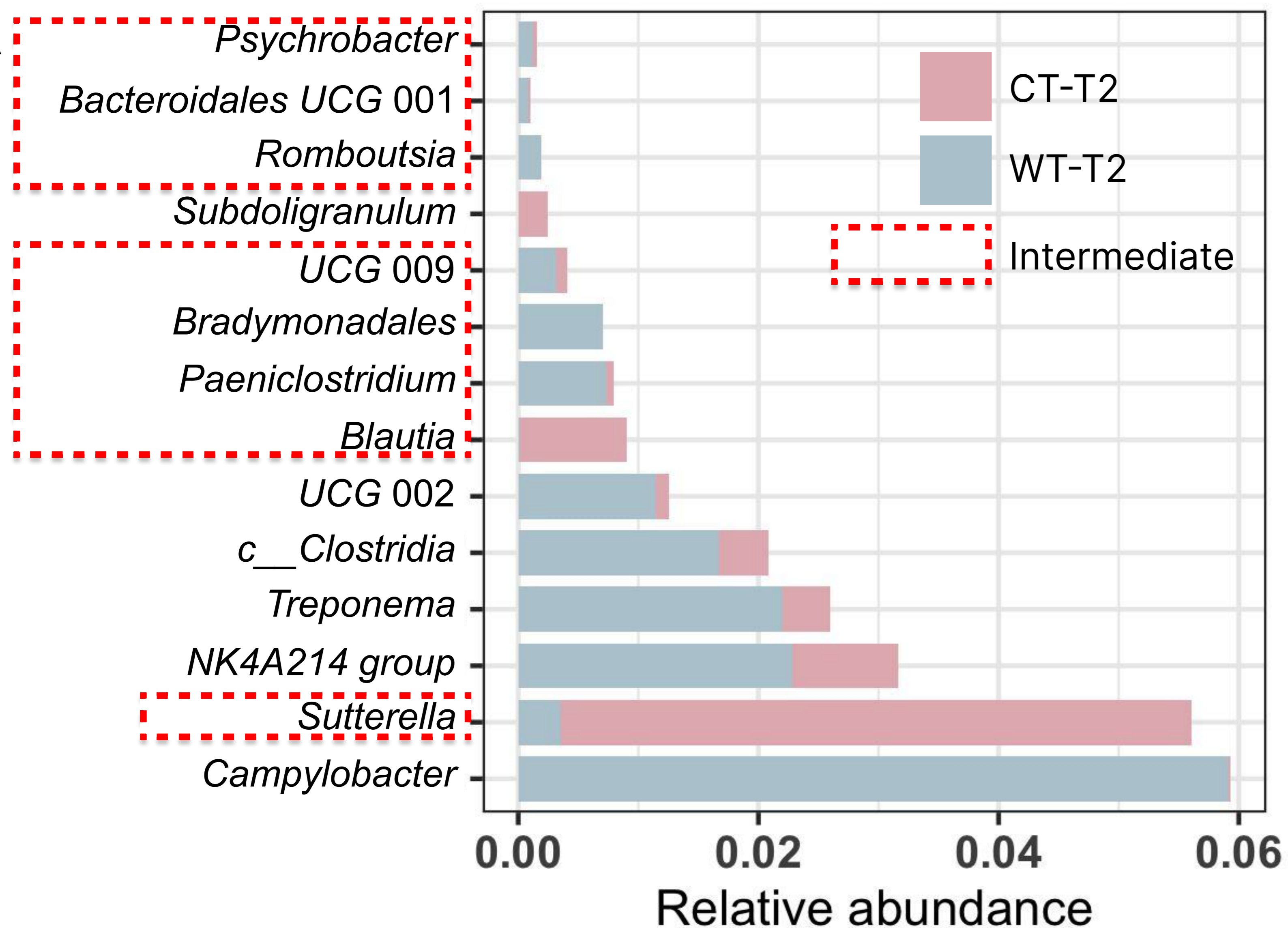

B

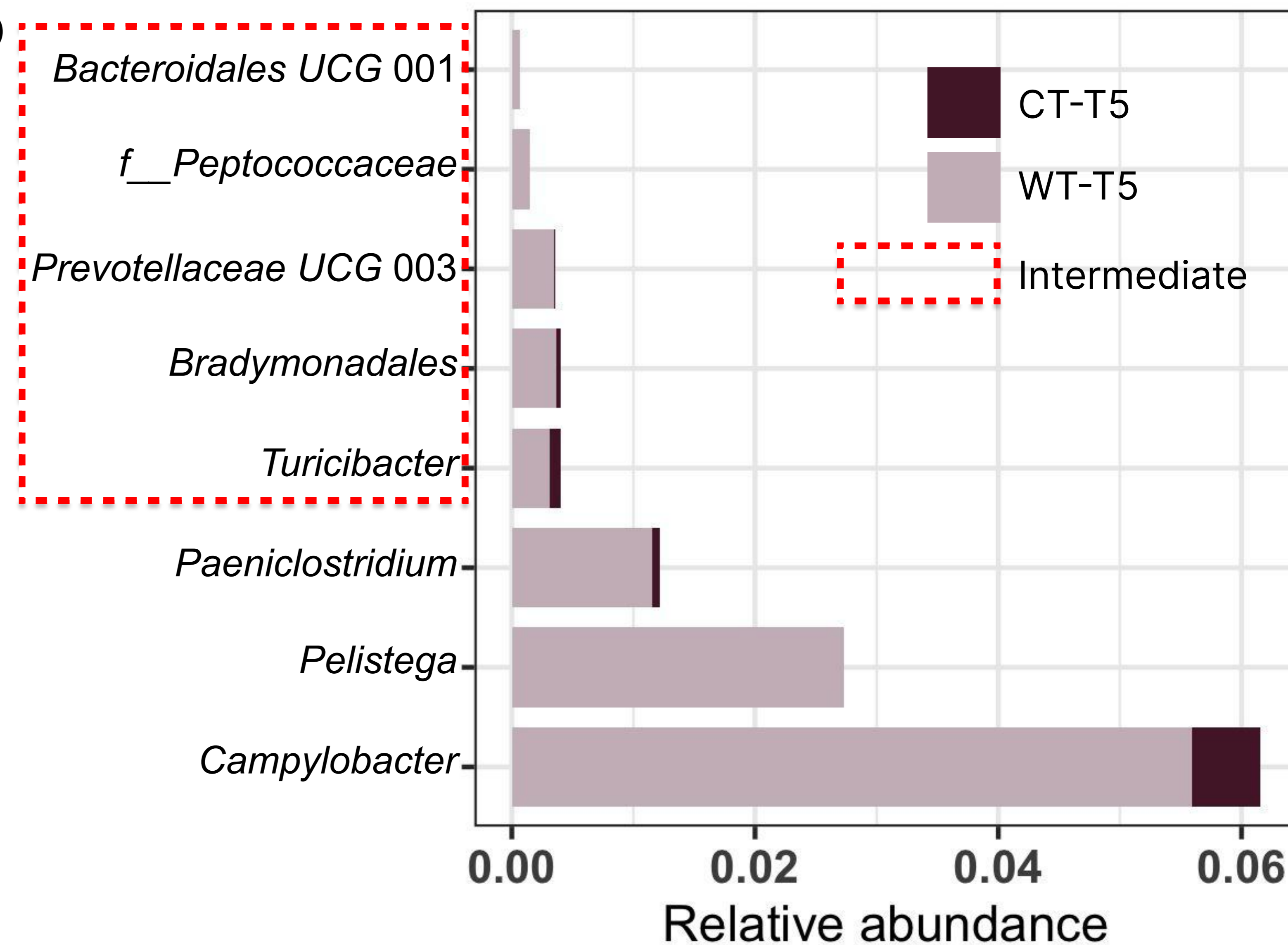

C

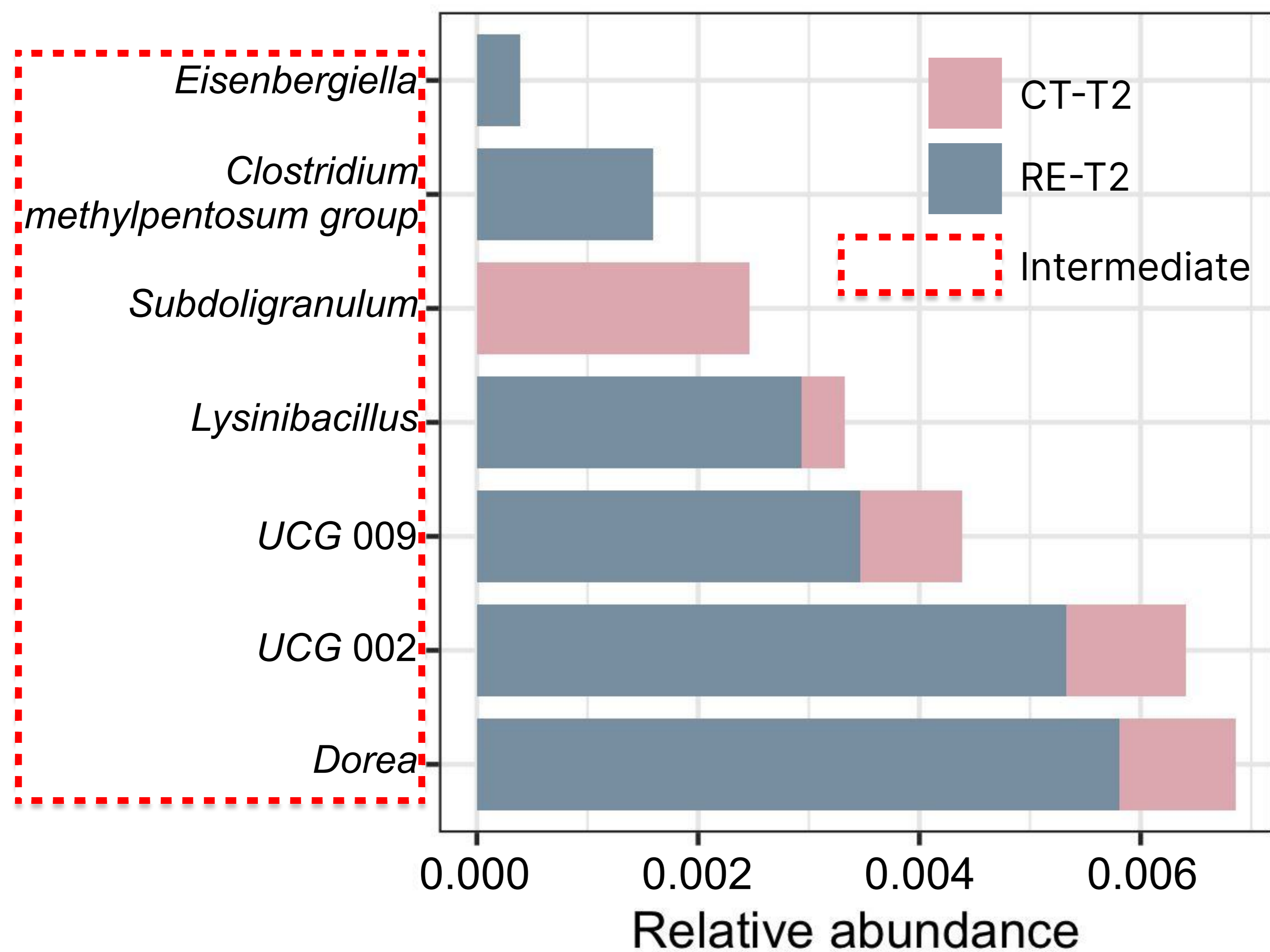

D

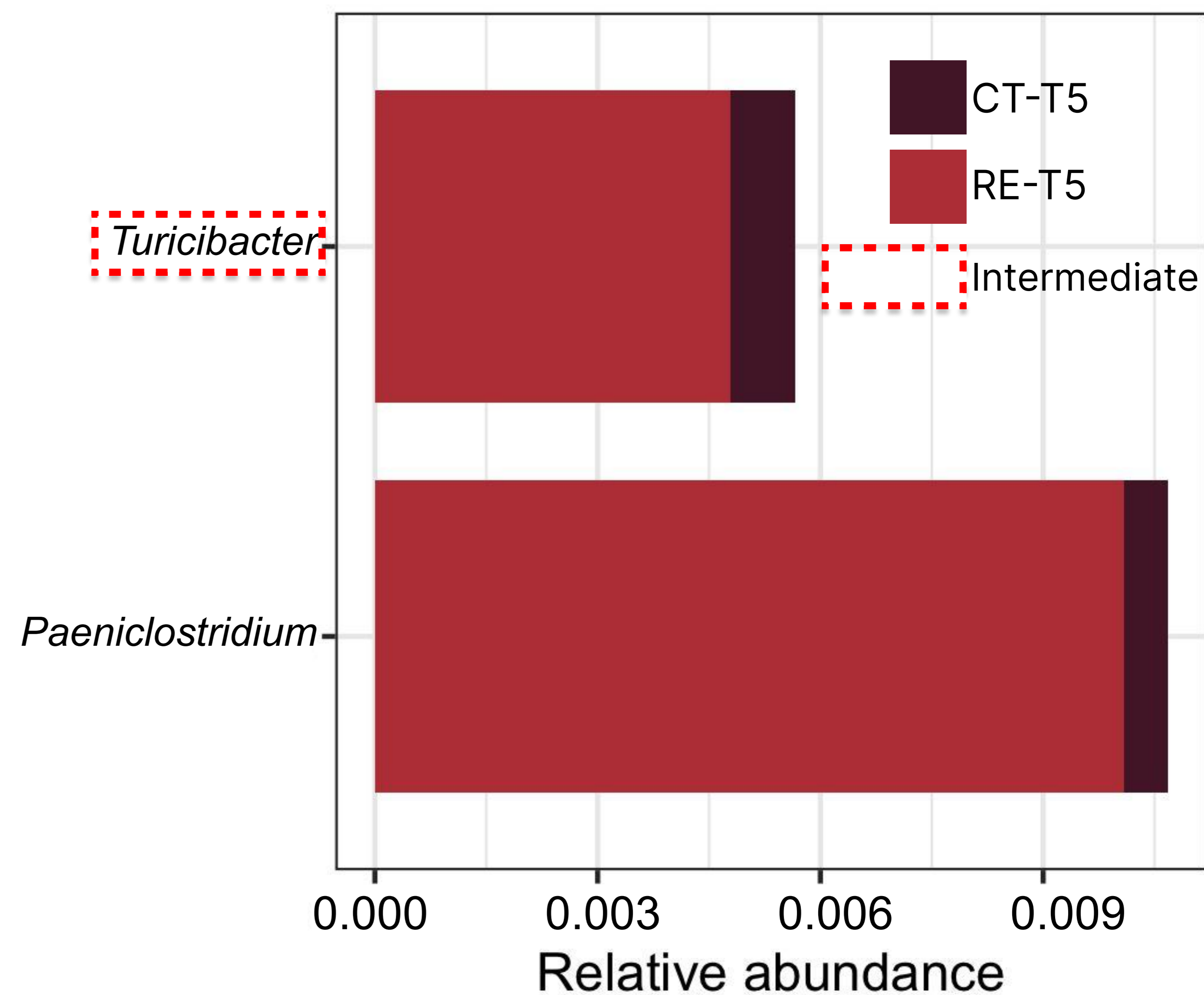

Supplement: Supplementary file 4 — Supplementary Material 3: Differential abundant microbes identified for WT-T2 vs CT-T2 (A), WT-T5 vs. CT-T5 (B), CT-T2 vs. RET2 (C), and CT-T5 vs. RE-T5 (D). The dashed rectangle represents the intermediate microbes. [file 40168_2025_2184_MOESM3_ESM.pdf]

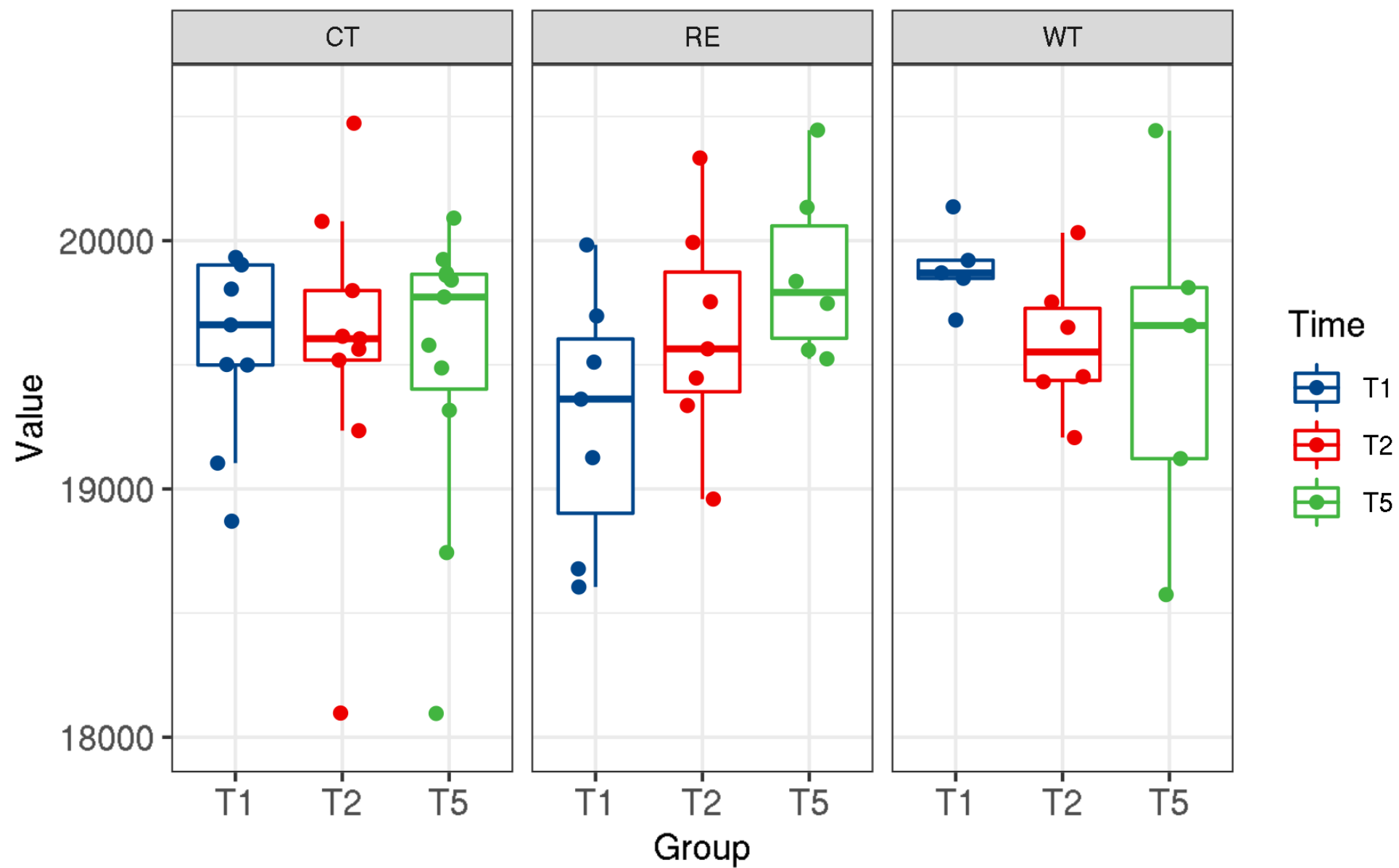

Supplement: Supplementary file 5 — Supplementary Material 4: The quantity of identified transcripts for CT, WT, and RE from T1 to T5. [file 40168_2025_2184_MOESM4_ESM.pdf]

A

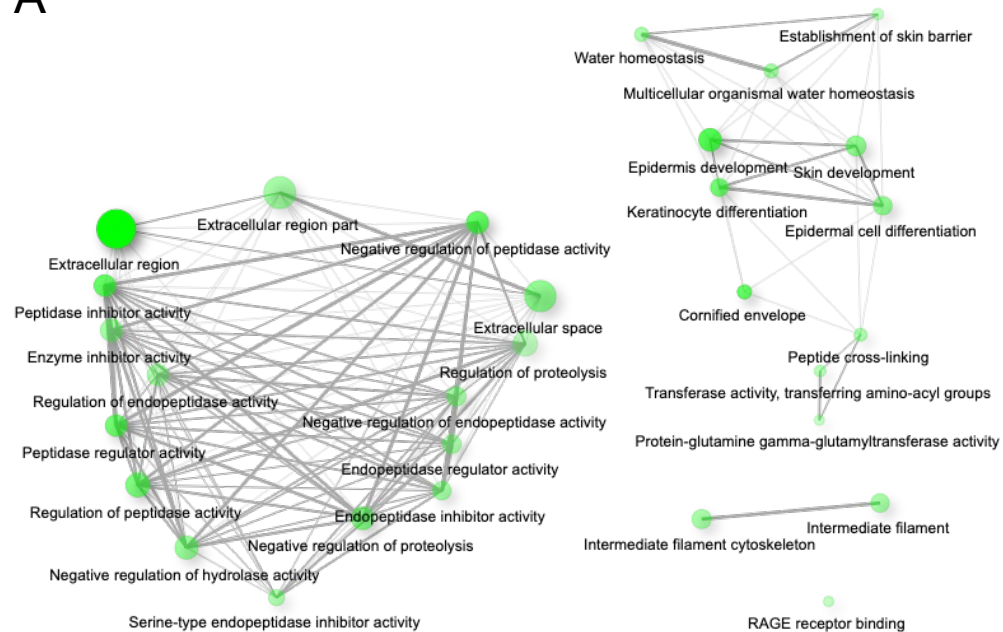

B

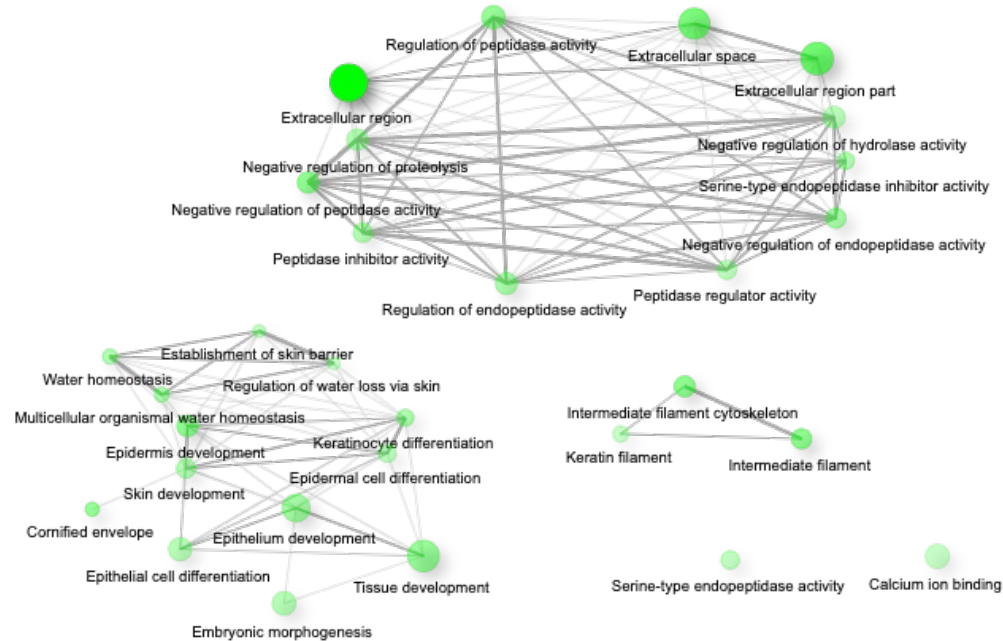

Supplement: Supplementary file 6 — Supplementary Material 5: The network showing relationships between enriched GO terms using DEGs at WT-T2 (A) and WT-T5 (B). Two pathways (nodes) are connected if they share 20% or more genes. Darker nodes are more significantly enriched gene sets. Bigger nodes represent larger gene sets. Thicker edges represent more overlapped genes. [file 40168_2025_2184_MOESM5_ESM.pdf]

A

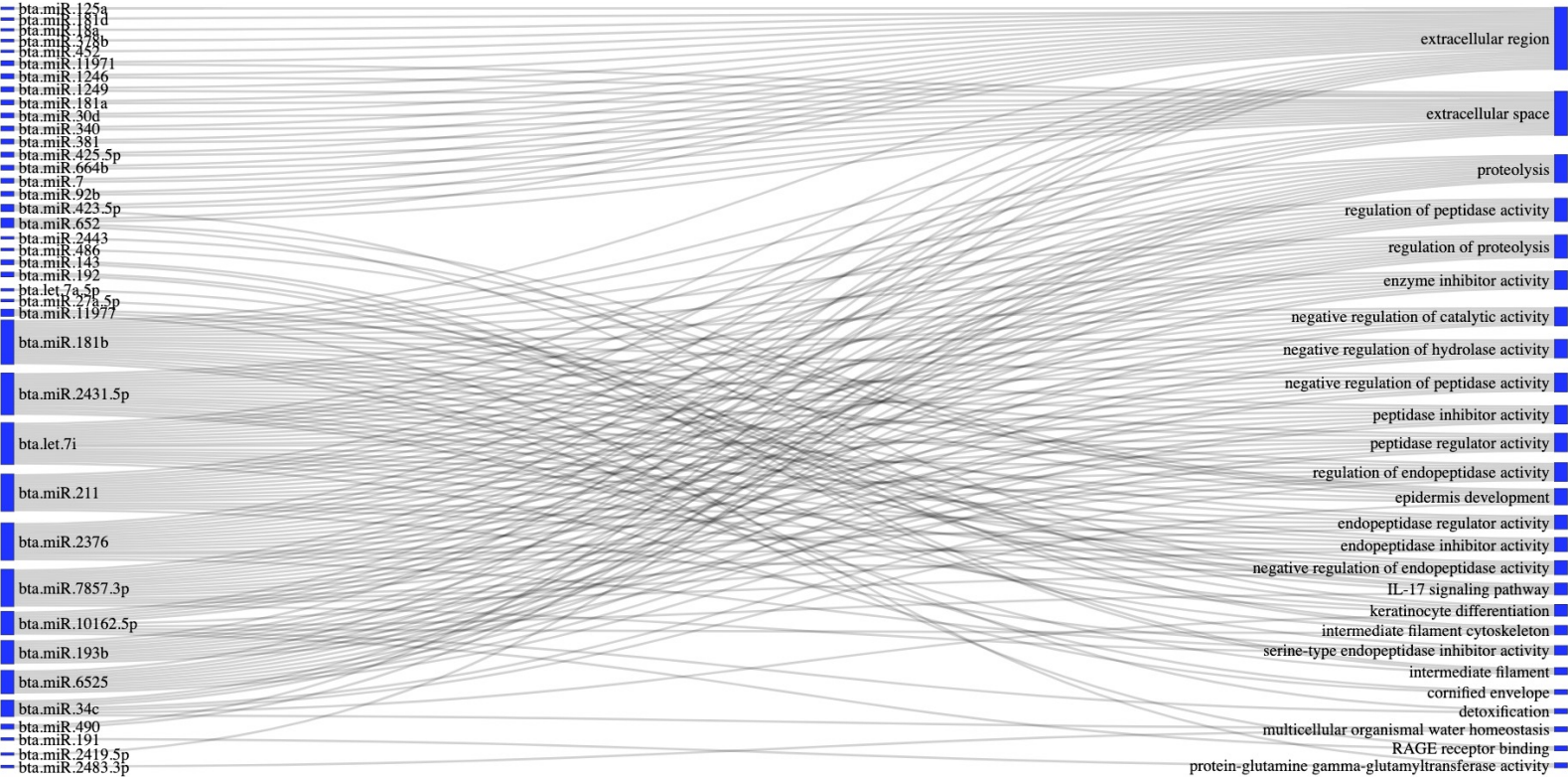

B

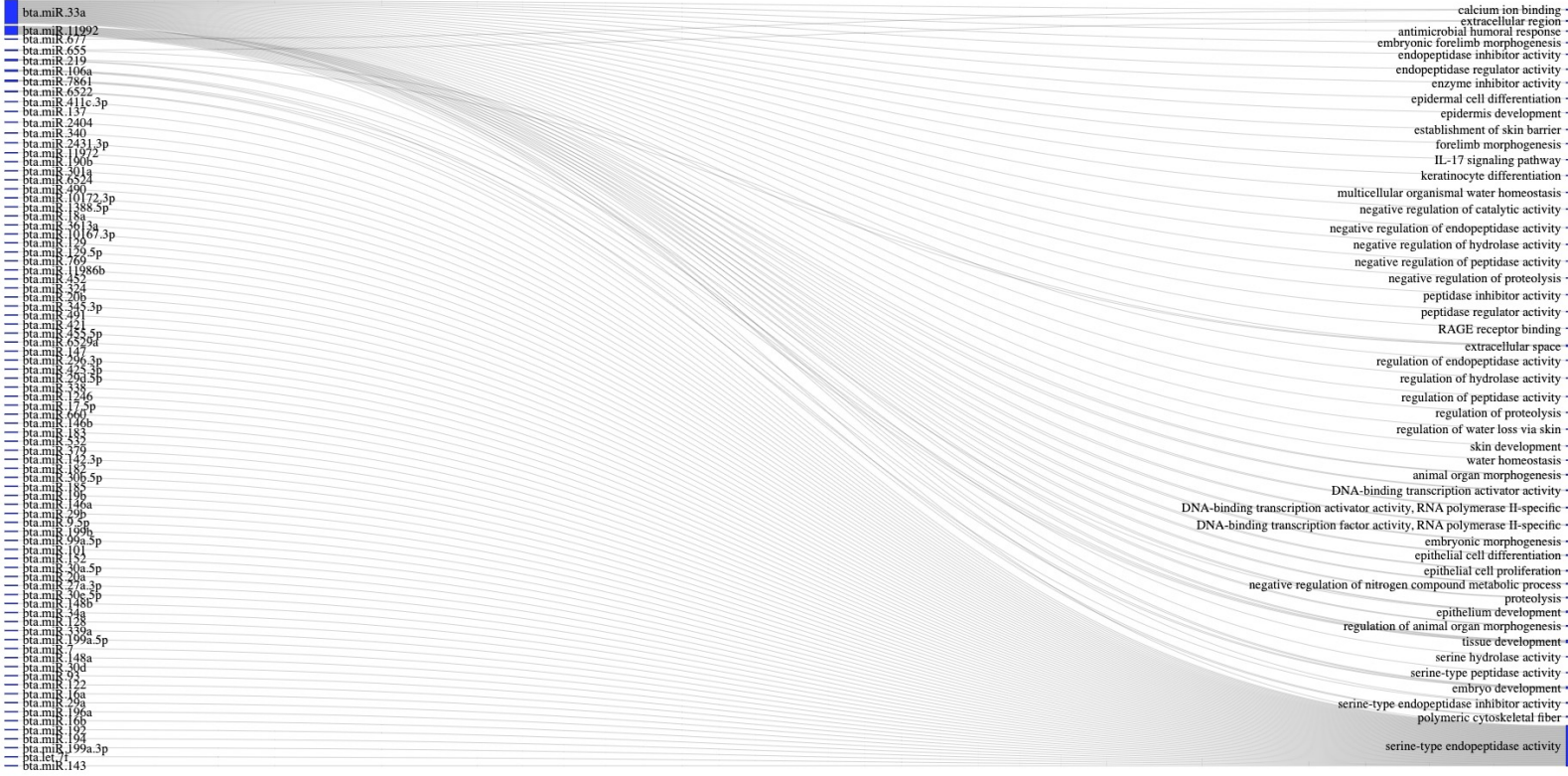

C

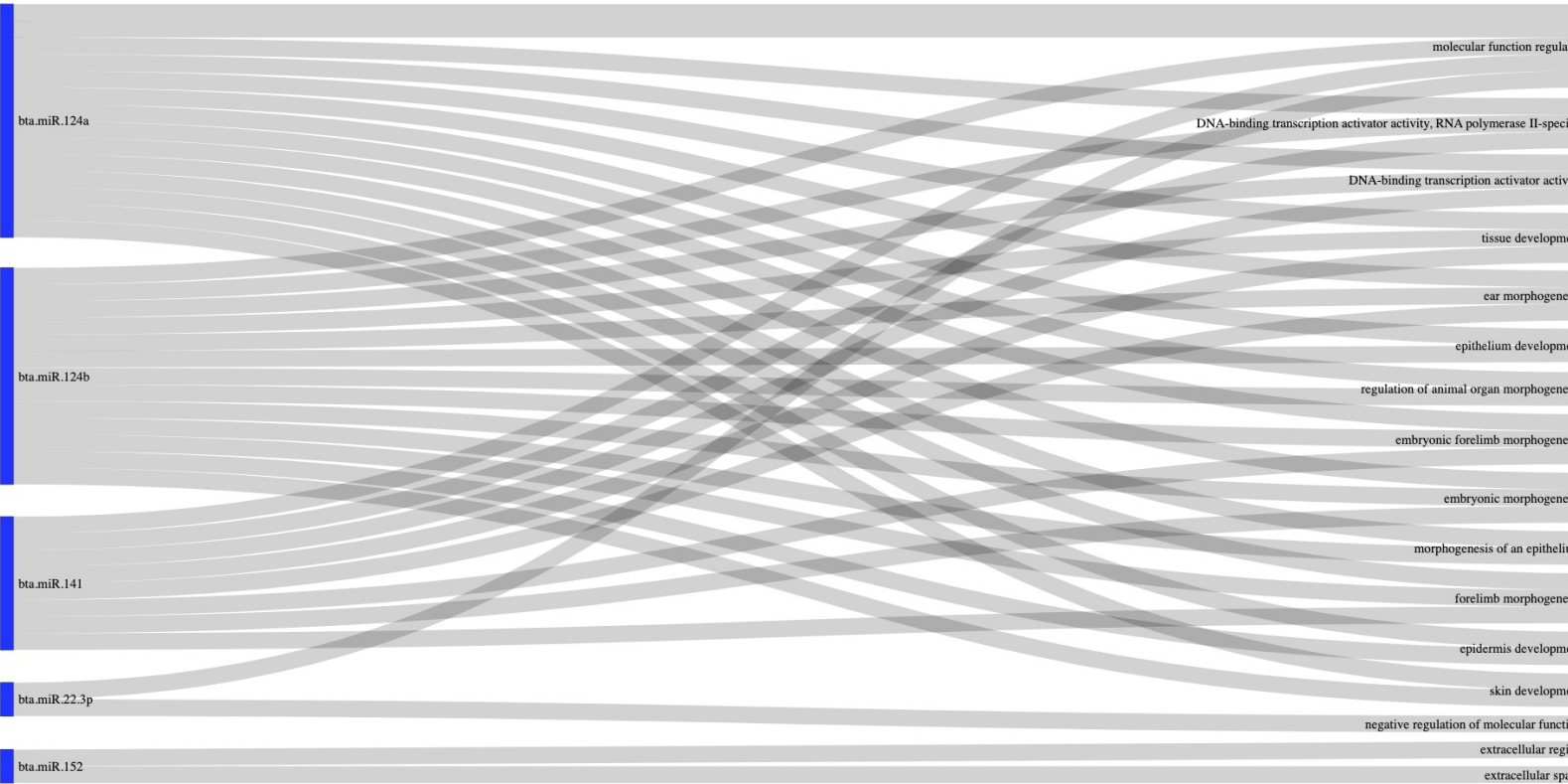

Supplement: Supplementary file 7 — Supplementary Material 6: The Sankey plot showing relationships between predicted miRNAs and functions enriched by differentially expressed genes at WT-T2 (A), WT-T5 (B), and RE-T2 (C) [file 40168_2025_2184_MOESM6_ESM.pdf]

A: WTT2

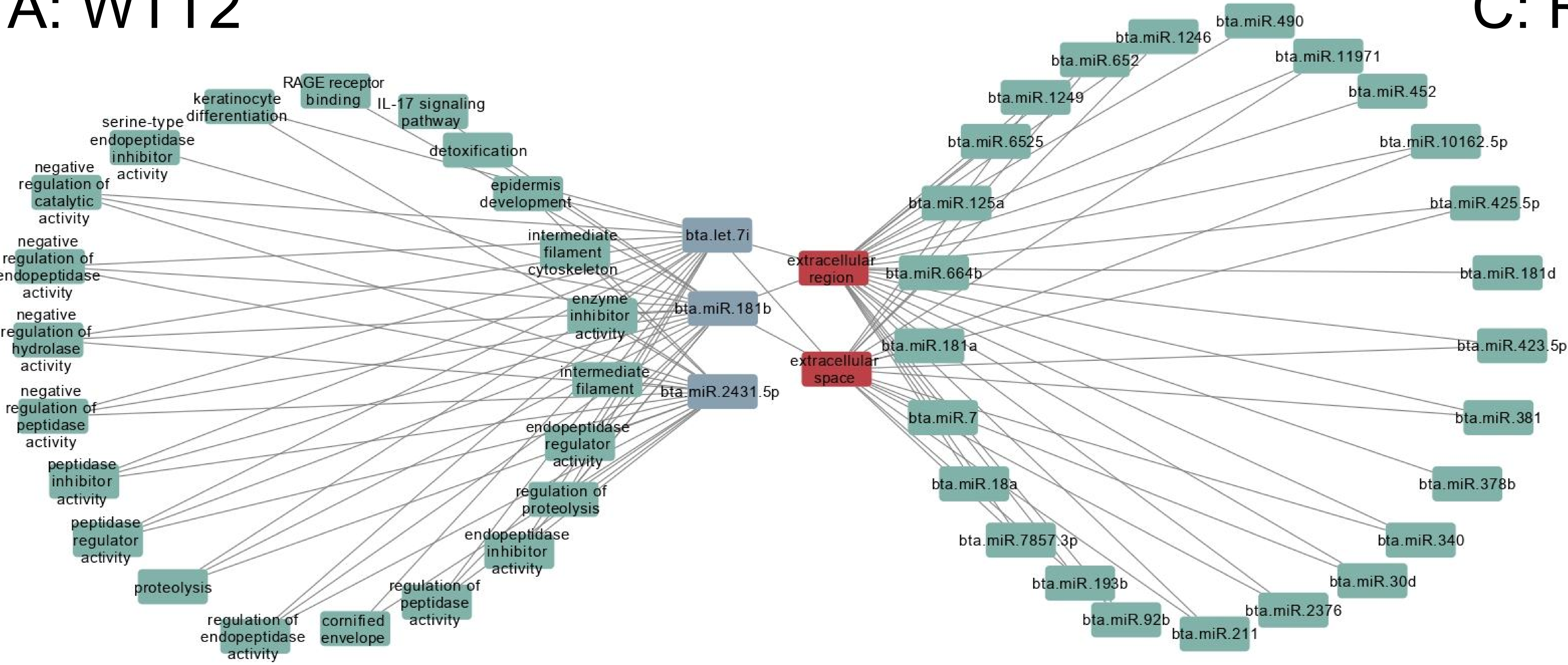

C: RET5

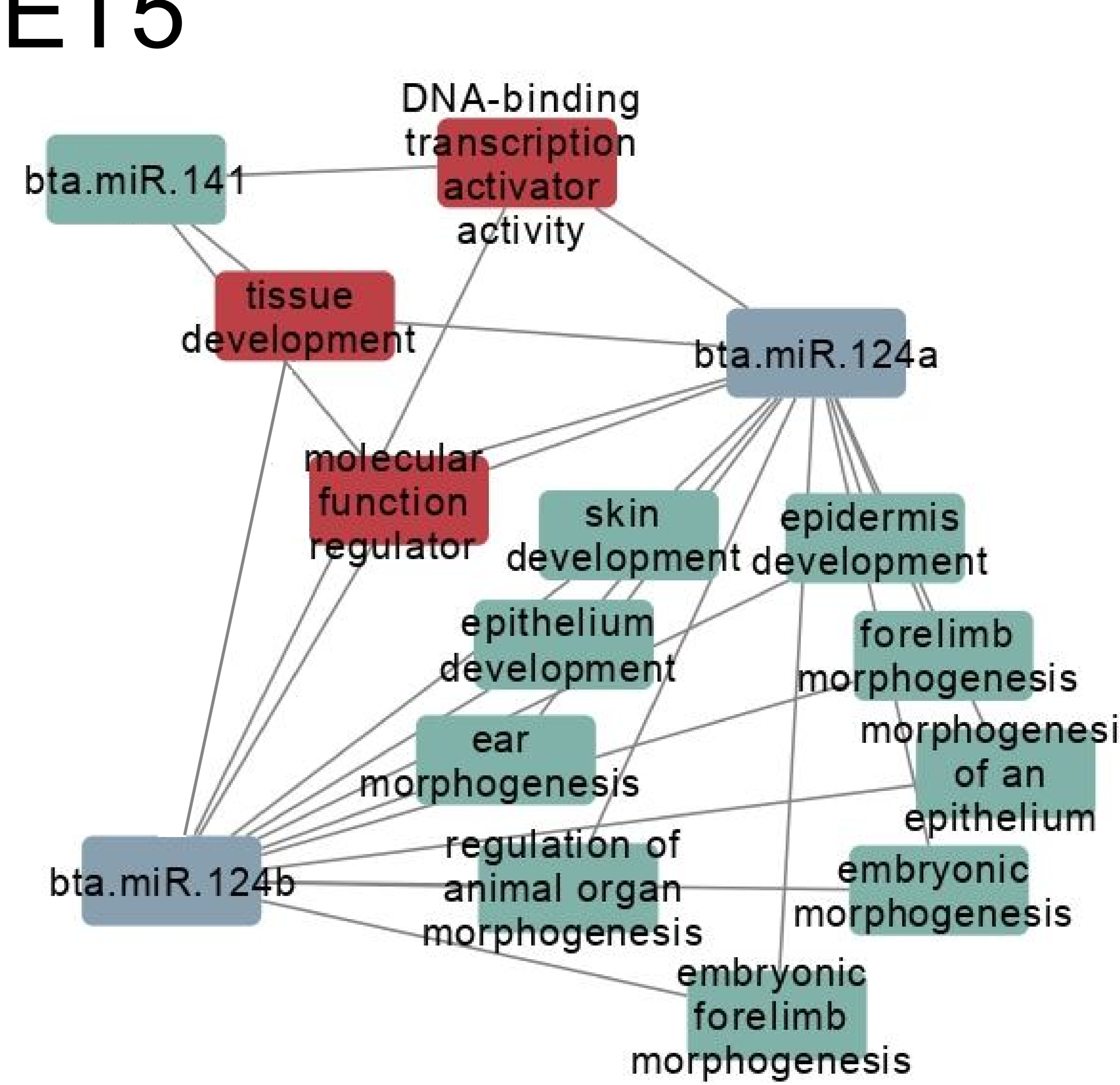

B: WTT5

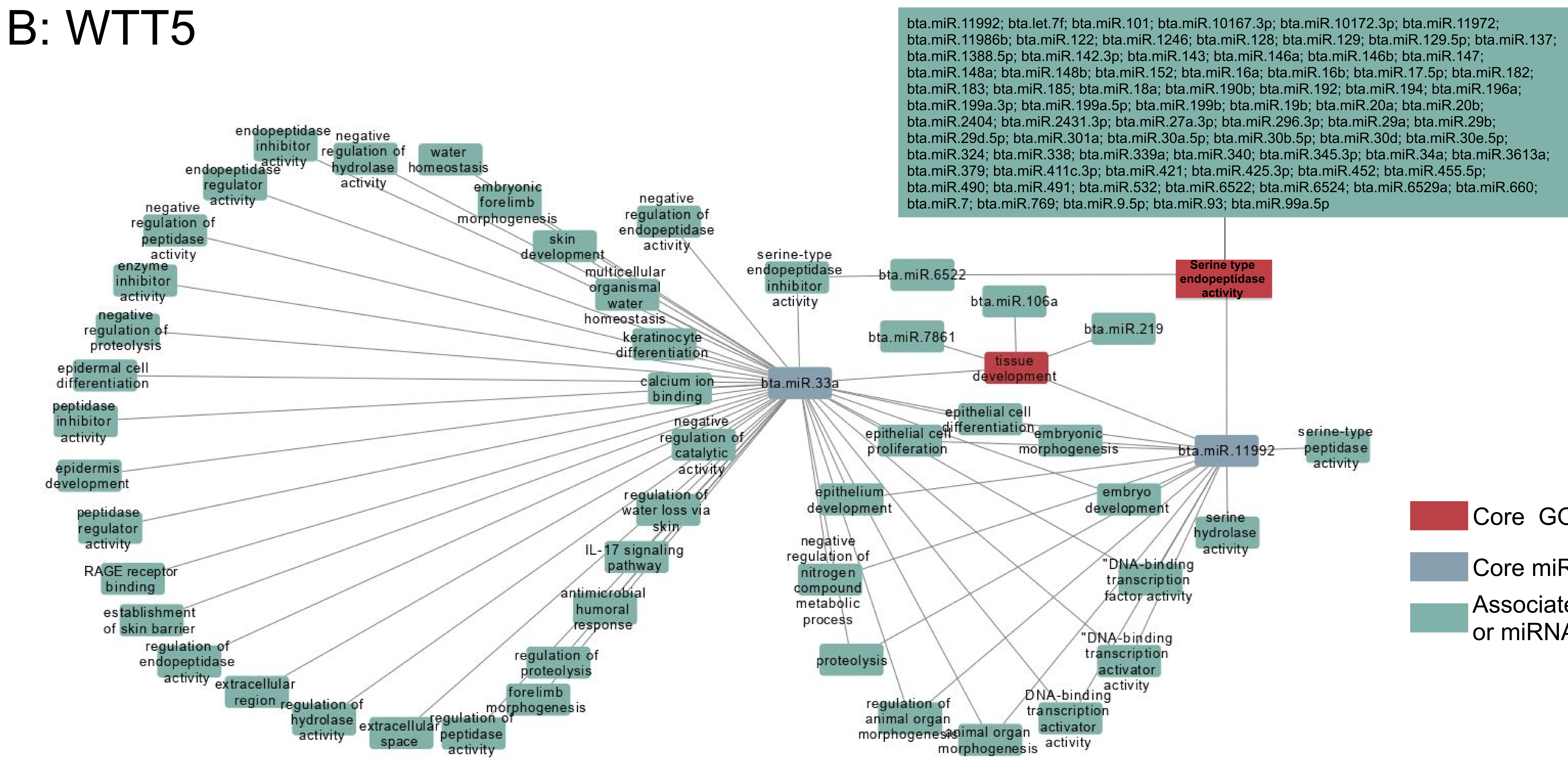

Core GO functions

Core miRNAs

Associated functions or miRNAs

Supplement: Supplementary file 8 — Supplementary Material 7: The interaction network between the most connected predicted miRNAs (Core miRNAs) and functions enriched by differentially expressed genes (Core GO functions) at WT-T2 (A), WT-T5 (B), RE-T5 (C) [file 40168_2025_2184_MOESM7_ESM.pdf]

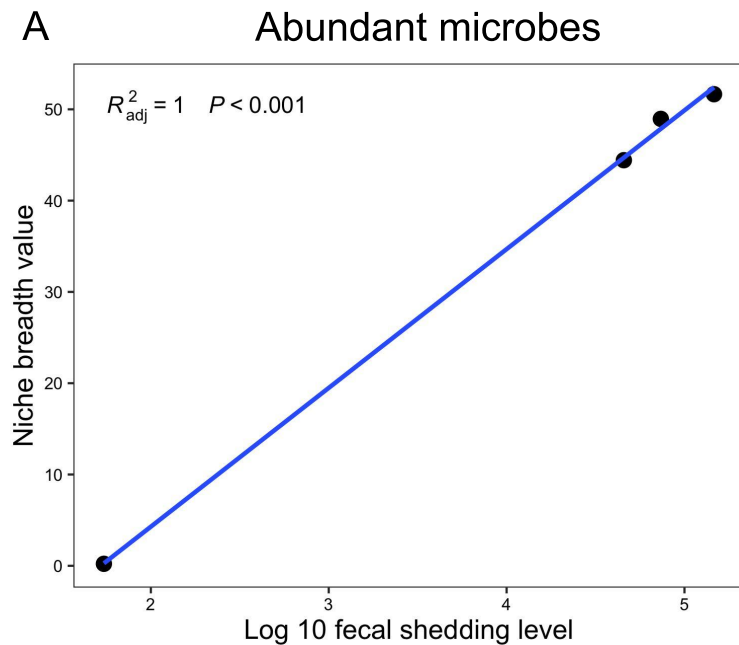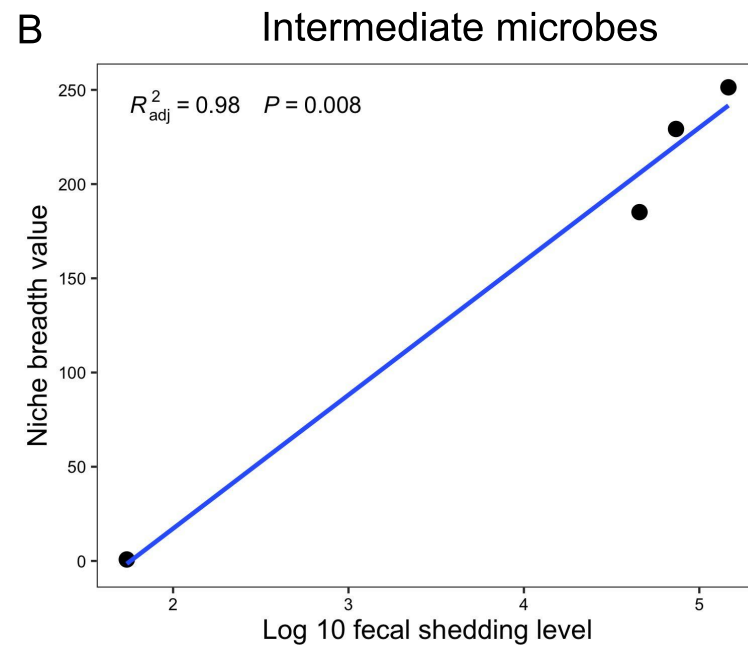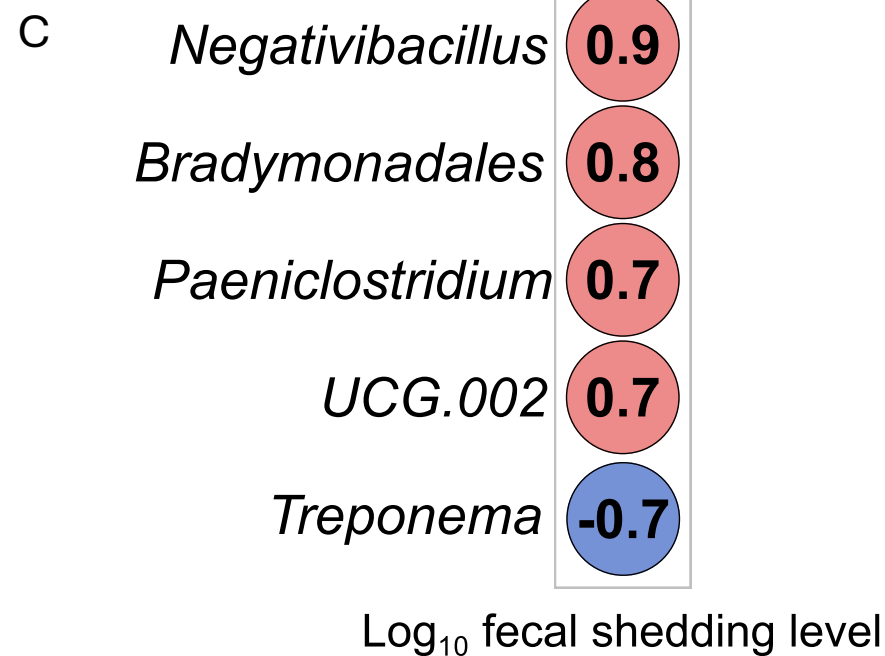

Supplement: Supplementary file 9 — Supplementary Material 8: Relationships between mucosa-attached microbes and log10 fecal shedding level. The linear regression plot showing relationships between log10 fecal shedding level and niche breadth values occupied by abundant (A) and intermediate microbes (B). The identified significant Spearman correlations between differential abundant microbes and log10 fecal shedding level at WT-T2 (C). The log‐‐ CFU values used were the group averages for WT-T2, WT-T5, RE-T2, and RE-T5. [file 40168_2025_2184_MOESM8_ESM.pdf]

Log10 fecal shedding level

WT-T2: bta-miR-190b

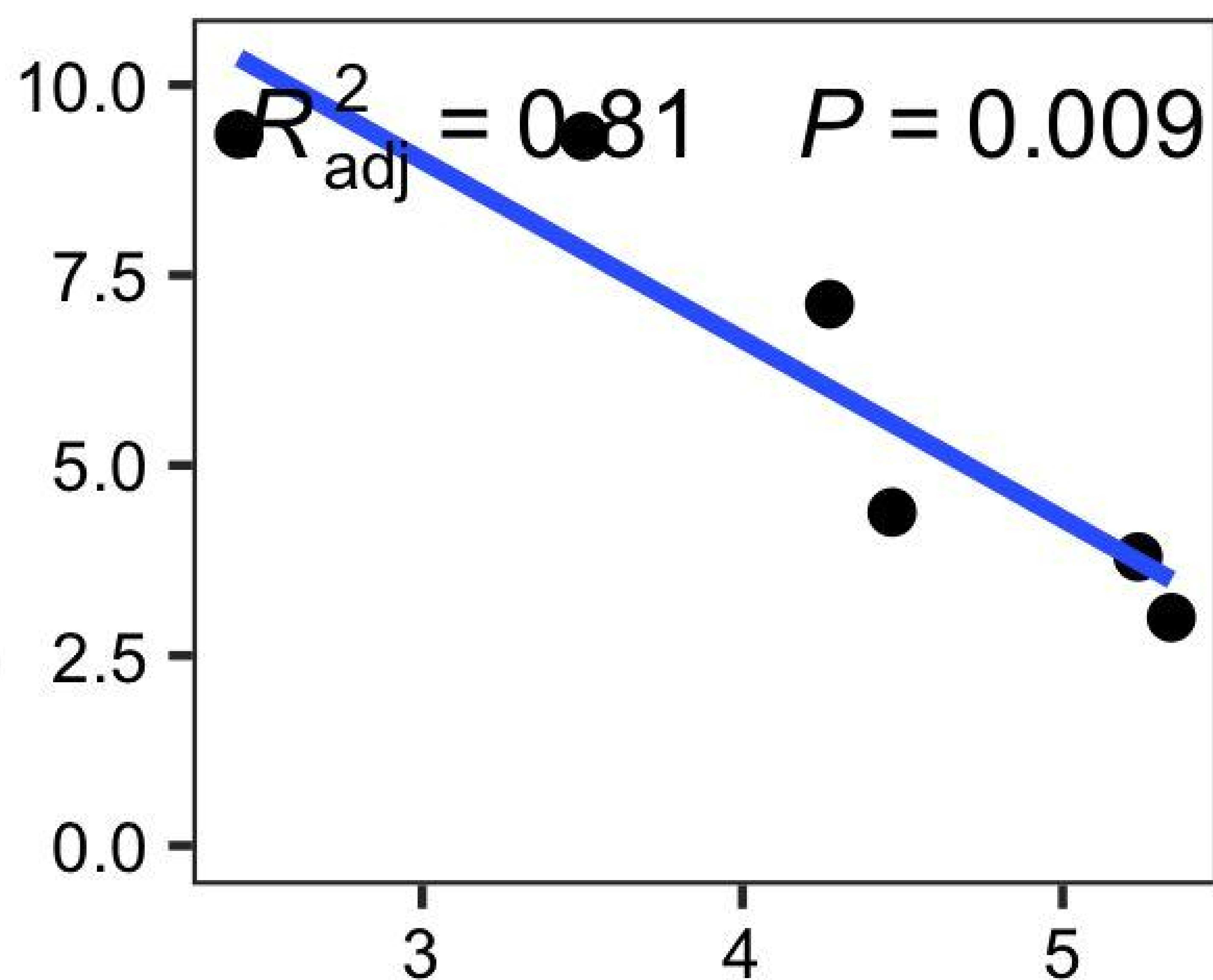

WT-T2: bta-miR-2285bt

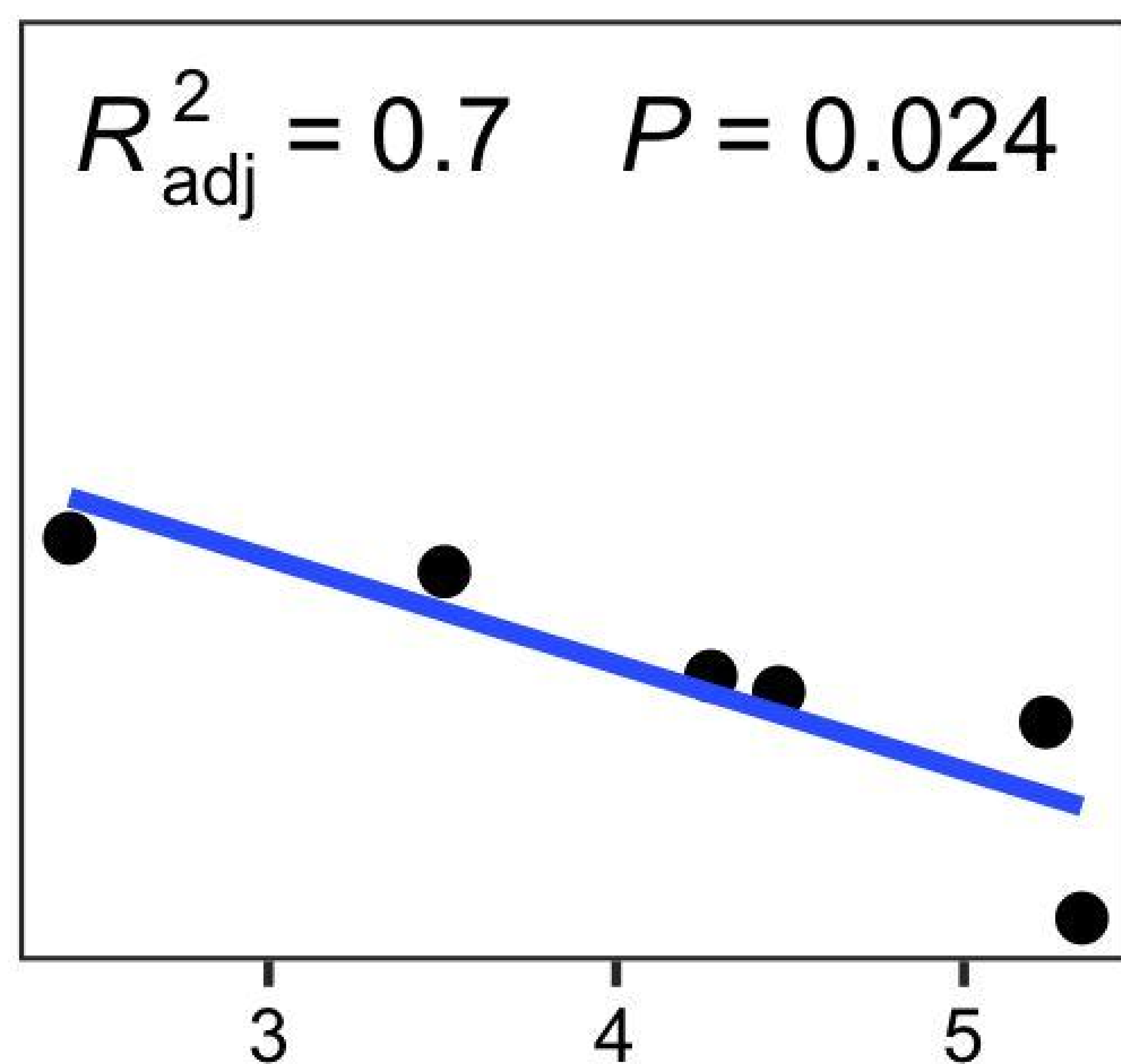

WT-T5: bta-miR-2285u

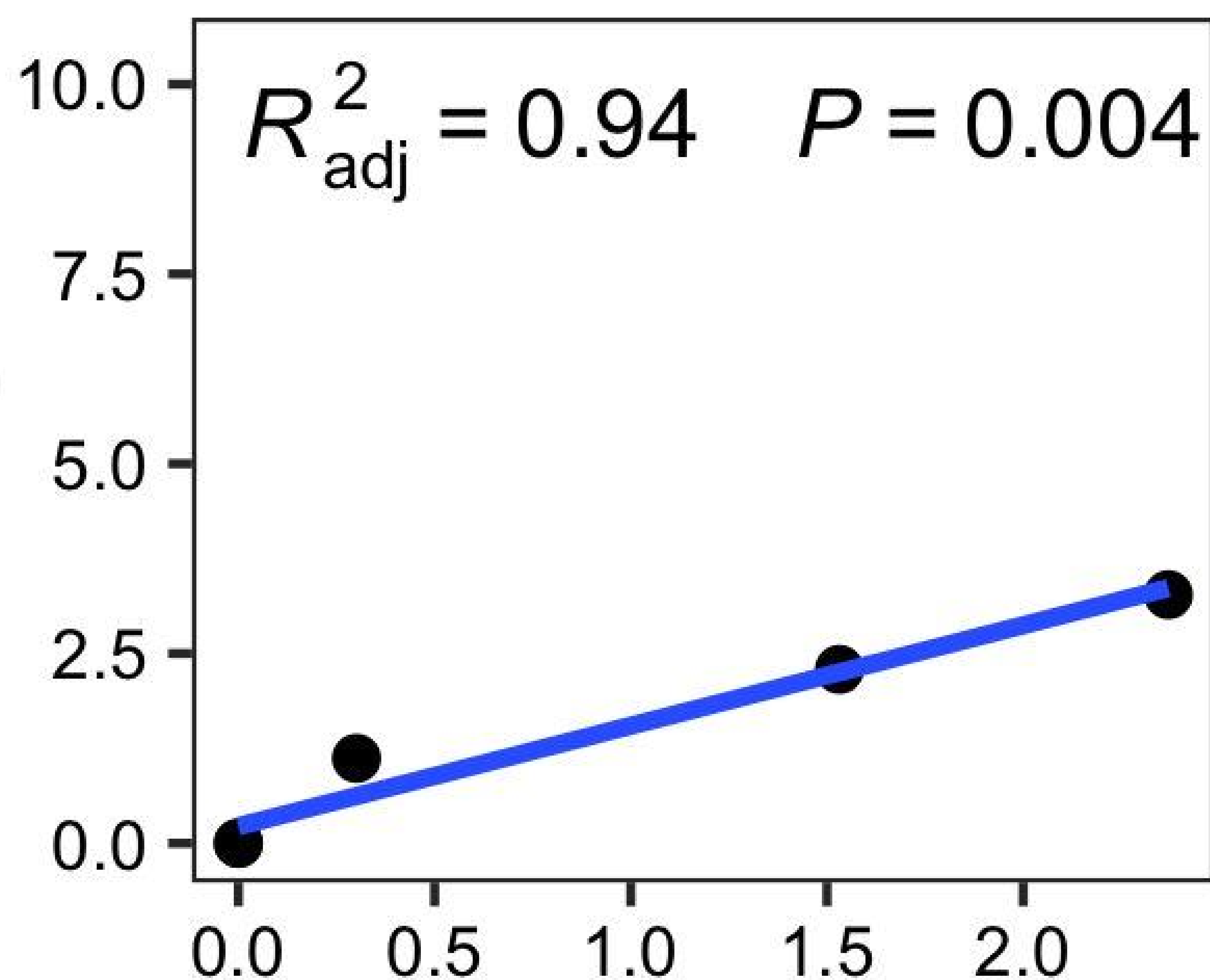

RE-T2: bta-miR-376e

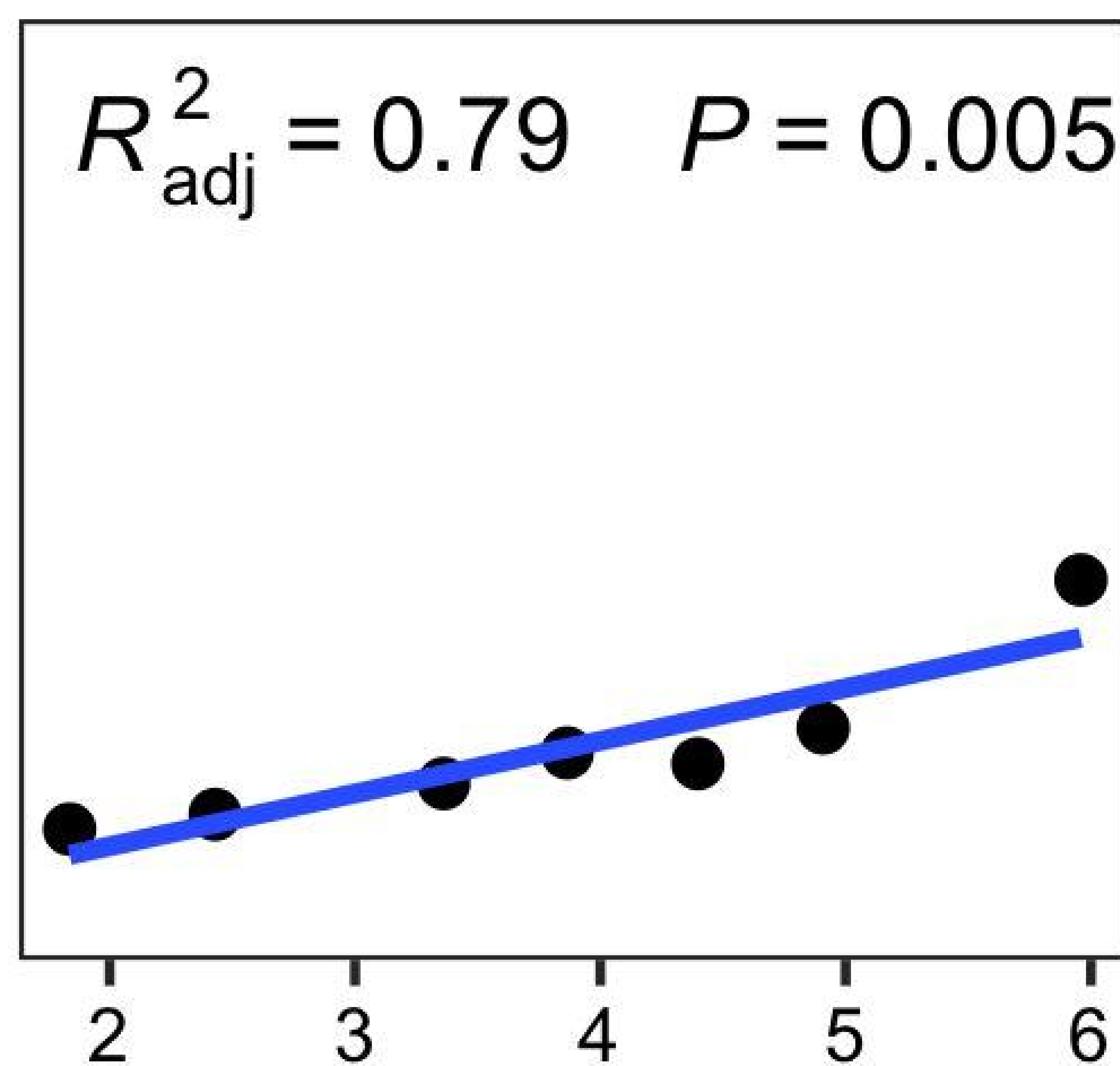

CPM

Supplement: Supplementary file 10 — Supplementary Material 9: The linear regression plot showing significant relationships between the CPM of miRNAs and log10 fecal shedding level. [file 40168_2025_2184_MOESM9_ESM.pdf]

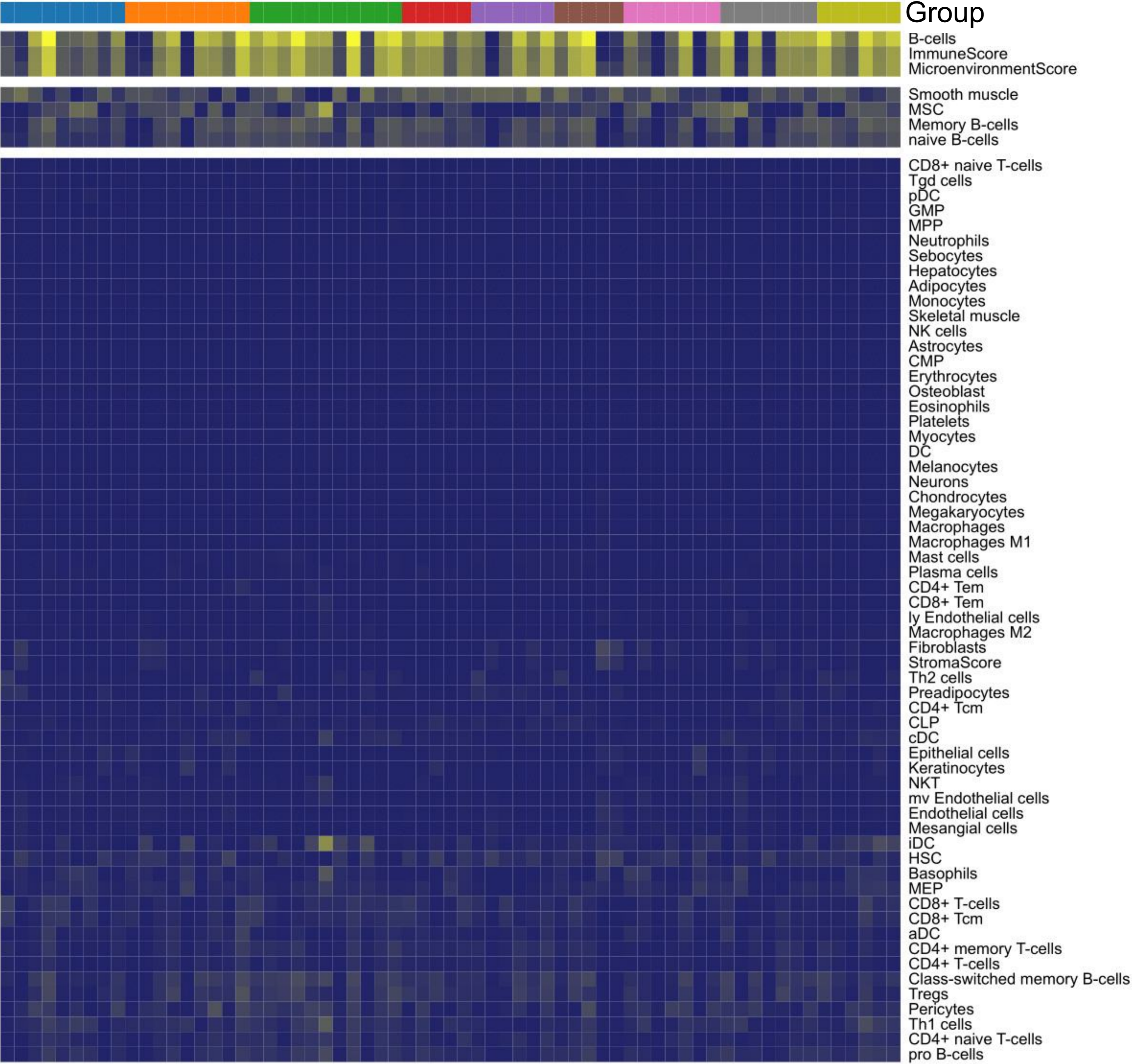

Supplement: Supplementary file 11 — Supplementary Material 10: The heatmap showing the xCell enrichment value for CT, WT, and RE from T1 to T5. [file 40168_2025_2184_MOESM10_ESM.pdf]

A

WT-T1

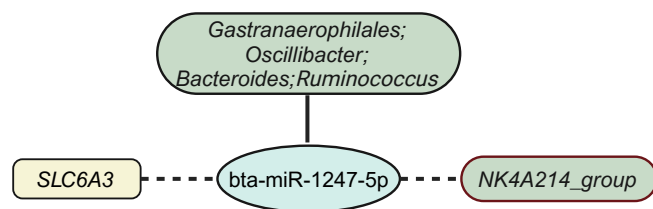

B

WT-T2

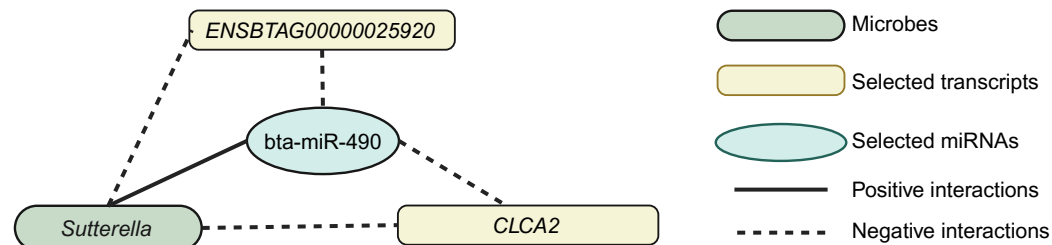

C

WT-T5

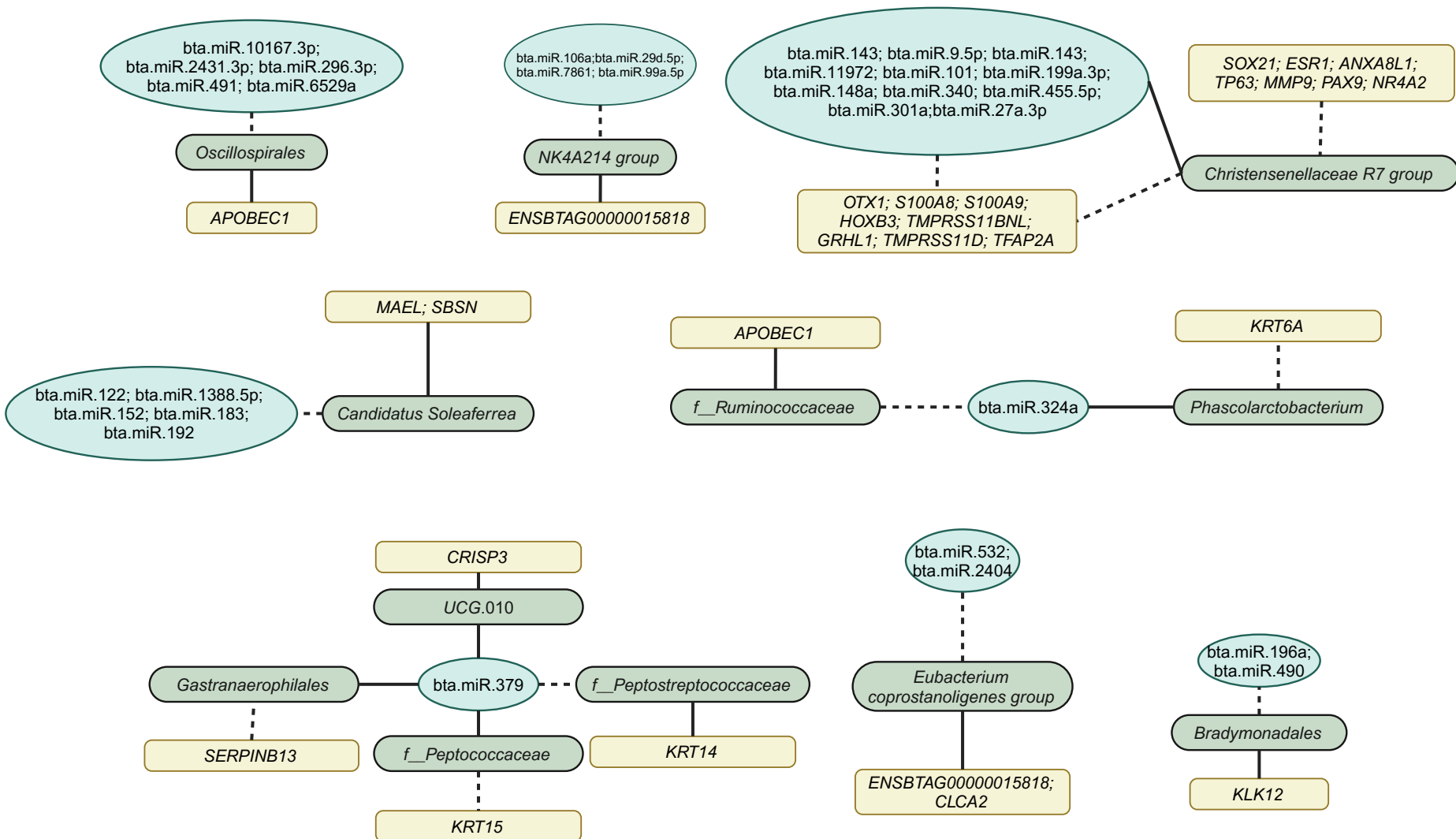

Supplement: Supplementary file 12 — Supplementary Material 11. The host miRNAs regulated host-microbiome interactions for WT-T1 (A), WT-T2 (B), and WT-T5 (C). The oval, rod, and rectangle shapes refer to miRNAs, microbes, and transcripts, respectively. The solid and dotted lines refer to positive and negative interactions, respectively. [file 40168_2025_2184_MOESM11_ESM.pdf]
