# Supplementary material for: Host miRNAs regulate Escherichia coli O157 mucosal colonization through host-mucosa-attached microbiota interactions in calves
Source: Microbiome. 2025 Oct 23;13:213. doi: 10.1186/s40168-025-02184-w (PMC12548129; doi:10.1186/s40168-025-02184-w)
Supplement: Supplementary file 13 — Supplementary Material 12. [file 40168_2025_2184_MOESM12_ESM.docx]

**Table S1.** The stratification of abundant-specific genera across each group. Fisher’s exact test was used due to low counts (i.e., fewer than 5) in multiple cells.

| Abundance | Group | Week | | | χ^2^ test | |
| --- | --- | --- | --- | --- | --- | --- |
|  |  | T1 | T2 | T5 | χ^2^ | p |
| Abundant | CT | 0.08 (n=11) | 0.070 (n=12) | 0.081 (n=12) | 1.1 | 0.9 |
|  | WT | 0.10 (n=11) | 0.092 (n=11) | 0.18 (n=18) |  |  |
|  | RE | 0.094 (n=11) | 0.097 (n=12) | 0.13 (n=13) |  |  |
| Intermediate | CT | 0.78 (n=104) | 0.82 (n=134) | 0.82 (n=122) | 4.1 | 0.4 |
|  | WT | 0.89 (n=93) | 0.90 (n=106) | 0.82 (n=80) |  |  |
|  | RE | 0.89 (n=104) | 0.90 (n=111) | 0.87 (n=85) |  |  |
| Rare | CT | 0.14 (n=18) | 0.11 (n=18) | 0.01 (n=14) | N/A | 0.8 |
|  | WT | 0.010 (n=1) | 0.017 (n=2) | 0 (n=0) |  |  |
|  | RE | 0.016 (n=2) | 0.0080 (n=1) | 0 (n=0) |  |  |

**Table S2.** Downregulated DEGs involved in host-immune related functions.

| Genes | Log2 fold change | P-value | Terms |
| --- | --- | --- | --- |
| *NOS2* | -1.5 | 0.029 | Defense response to bacterium |
| *LPO* | -7.3 | <0.001 |  |
| *LBP* | -2.1 | 0.005 |  |
| *DEFB10* | -1.7 | 0.021 |  |
| *ENSBTAG00000052579* | -2.3 | 0.019 |  |
| *EVPL* | -2.2 | 0.040 | Antimicrobial humoral response & Antibacterial humoral response & Defense response to bacterium |
| *ENSBTAG00000014329* | -4.7 | 0.009 |  |
| *ENSBTAG00000046944* | -1.9 | 0.005 |  |
| *S100A9* | -5.7 | <0.001 | Antimicrobial humoral response & Humoral immune response & Antimicrobial humoral immune response mediated by antimicrobial peptide |
| *S100A12* | -3.4 | 0.014 |  |
| *GRO1* | 2.3 | <0.001 | Humoral immune response & Antimicrobial humoral immune response mediated by antimicrobial peptide |
| *PGLYRP4* | -2.7 | 0.018 |  |
| *SLPI* | 5.4 | 0.005 | Humoral immune response |
| *KRT1* | -6.7 | <0.001 |  |
| *SPINK5* | -7.9 | <0.001 | Antimicrobial humoral response & Antibacterial humoral response & Defense response to bacterium & Humoral immune response & Antimicrobial humoral immune response mediated by antimicrobial peptide |
| *WFDC5* | -23.9 | <0.001 | Antimicrobial humoral response & Antibacterial humoral response & Defense response to bacterium & Humoral immune response |

**Table S3.** The average number of identified miRNAs for CT, WT, and RE from T1 to T5.

| Group | Time point | The average number of identified miRNAs | Standard deviation |
| --- | --- | --- | --- |
| CT | T1 | 406 | 60 |
|  | T2 | 425 | 15 |
|  | T5 | 436 | 19 |
| WT | T1 | 414 | 12 |
|  | T2 | 428 | 19 |
|  | T5 | 431 | 19 |
| RE | T1 | 424 | 18 |
|  | T2 | 429 | 22 |
|  | T5 | 416 | 15 |

**Table S4.** Identification of differential expressed host miRNAs in comparison of WT vs. CT, RE vs. CT and WT vs. RE from T1 to T5.

| Group | Time | Up-regulated miRNAs | Down-regulated miRNAs |
| --- | --- | --- | --- |
| WT vs. CT | T1 | 1 | 1 |
|  | T2 | / | / |
|  | T5 | 4 | 1 |
| RE vs. CT | T1 | / | / |
|  | T2 | 2 | 0 |
|  | T5 | / | / |
| WT vs. RE | T1 | 2 | 1 |
|  | T2 | 1 | 13 |
|  | T5 | 1 | 5 |

**Table S5.** The list of differential expressed host miRNAs in comparison of WT vs. CT, RE vs. CT and WT vs. RE from T1 to T5.

| Group of comparison | Time point | DE miRNAs | Log2 fold change | P adj |
| --- | --- | --- | --- | --- |
| WT vs CT | T1 | bta-miR-1247-5p | -1.78 | 0.03 |
|  |  | bta-miR-101 | 1.56 | 0.03 |
|  | T5 | bta-miR-211 | -2.09 | <0.01 |
|  |  | bta-miR-184 | 2.20 | <0.01 |
|  |  | bta-miR-2311 | 2.25 | 0.01 |
|  |  | bta-miR-2887 | 2.09 | 0.01 |
|  |  | bta-miR-2440 | 1.90 | 0.03 |
| RE vs CT | T2 | bta-miR-219 | 1.80 | 0.03 |
|  |  | bta-miR-1224 | 1.60 | 0.04 |
| WT vs RE | T1 | bta-miR-101 | 1.80 | <0.01 |
|  |  | bta-miR-142-3p | 1.79 | <0.01 |
|  |  | bta-miR-760-3p | -1.53 | 0.04 |
|  | T2 | bta-miR-2285bh | 1.74 | 0.01 |
|  |  | bta-miR-744 | -1.54 | <0.01 |
|  |  | bta-miR-874 | -1.55 | <0.01 |
|  |  | bta-miR-6517 | -1.55 | <0.01 |
|  |  | bta-miR-10179-5p | -1.56 | 0.03 |
|  |  | bta-miR-11976 | -1.56 | 0.01 |
|  |  | bta-miR-3957 | -1.58 | 0.01 |
|  |  | bta-miR-10167-3p | -1.60 | 0.01 |
|  |  | bta-miR-485 | -1.64 | 0.01 |
|  |  | bta-miR-10182-5p | -1.69 | 0.02 |
|  |  | bta-miR-296-3p | -1.70 | 0.00 |
|  |  | bta-miR-502b | -1.74 | 0.01 |
|  |  | bta-miR-423-5p | -1.75 | <0.01 |
|  |  | bta-miR-2474 | -1.76 | 0.01 |
|  |  | bta-miR-760-3p | -1.78 | <0.01 |
|  |  | bta-miR-3533 | -1.78 | 0.01 |
|  |  | bta-miR-504 | -1.86 | 0.01 |
|  |  | bta-miR-1343-3p | -1.88 | 0.00 |
|  |  | bta-miR-11982 | -1.95 | 0.01 |
|  |  | bta-miR-1307 | -1.97 | <0.01 |
|  |  | bta-miR-1291 | -1.98 | 0.01 |
|  |  | bta-miR-365-5p | -2.05 | <0.01 |
|  |  | bta-miR-2440 | -2.09 | <0.01 |
|  |  | bta-miR-10185-5p | -2.20 | <0.01 |
|  |  | bta-miR-1224 | -2.22 | <0.01 |
|  |  | bta-miR-11980 | -2.31 | <0.01 |
|  | T5 | bta-miR-2311 | 2.01 | 0.02 |
|  |  | bta-miR-181c | -1.55 | <0.01 |
|  |  | bta-miR-362-3p | -1.67 | 0.05 |
|  |  | bta-miR-2285f | -1.88 | <0.01 |
|  |  | bta-miR-2285ce | -1.92 | 0.02 |
|  |  | bta-miR-2285bf | -2.01 | <0.01 |
